# Solution NMR Structure of the SH3 Domain of Human Caskin1 Validates the Lack of a Typical Peptide Binding Groove and Supports a Role in Lipid Mediator Binding

**DOI:** 10.3390/cells10010173

**Published:** 2021-01-16

**Authors:** Orsolya Tőke, Kitti Koprivanacz, László Radnai, Balázs Merő, Tünde Juhász, Károly Liliom, László Buday

**Affiliations:** 1 Laboratory for NMR Spectroscopy, Research Centre for Natural Sciences, 2 Magyar tudósok körútja, H-1117 Budapest, Hungary; 2 Institute of Enzymology, Research Centre for Natural Sciences, 2 Magyar tudósok körútja, H-1117 Budapest, Hungary; kitti.koprivanacz@gmail.com (K.K.); lradnai@gmail.com (L.R.); merobwork@gmail.com (B.M.); juhasz.tunde@ttk.hu (T.J.); 3 Department of Biophysics and Radiation Biology, Semmelweis University, 37-47 Tűzoltó utca, H-1094 Budapest, Hungary; karoly.liliom.mta@gmail.com

**Keywords:** caskin1, SH3 domain, lipid signaling, lysophosphatidic acid, protein-lipid-interaction, NMR spectroscopy, molecular recognition

## Abstract

SH3 domains constitute an important class of protein modules involved in a variety of cellular functions. They participate in protein-protein interactions via their canonical ligand binding interfaces composed of several evolutionarily conserved aromatic residues forming binding grooves for typical (PxxP) and atypical (PxxxPR, RxxK, RKxxY) binding motifs. The calcium/calmodulin-dependent serine protein kinase (CASK)-interacting protein 1, or Caskin1, a multidomain scaffold protein regulating the cortical actin filaments, is enriched in neural synapses in mammals. Based on its known interaction partners and knock-out animal studies, Caskin1 may play various roles in neural function and it is thought to participate in several pathological processes of the brain. Caskin1 has a single, atypical SH3 domain in which key aromatic residues are missing from the canonical binding groove. No protein interacting partner for this SH3 domain has been identified yet. Nevertheless, we have recently demonstrated the specific binding of this SH3 domain to the signaling lipid mediator lysophospatidic acid (LPA) in vitro. Here we report the solution NMR structure of the human Caskin1 SH3 domain and analyze its structural features in comparison with other SH3 domains exemplifying different strategies in target selectivity. The key differences revealed by our structural study show that the canonical binding groove found in typical SH3 domains accommodating proline-rich motifs is missing in Caskin1 SH3, most likely excluding a bona fide protein target for the domain. The LPA binding site is distinct from the altered protein binding groove. We conclude that the SH3 domain of Caskin1 might mediate the association of Caskin1 with membrane surfaces with locally elevated LPA content.

## 1. Introduction

The calcium/calmodulin-dependent serine protein kinase (CASK)-interacting protein 1 or Caskin1, enriched in neuronal synapses in mammals, is an adaptor protein regulating cortical actin filaments [1]. Similar to its isoform, Caskin2, it is a multidomain protein containing six ankyrin repeats, a single Src homology 3 (SH3) domain, two sterile α motif (SAM) domains, an intrinsically disordered proline-rich segment, and a unique C-terminal conserved region [1,2]. In a complex with CASK, Caskin1 associates with the cytoplasmic tail of neurexin1, a neuron specific adhesion molecule [1]. Regarding the CASK-Caskin1 interaction, a short linear motif in the unstructured linker connecting the SH3 and SAM domains of Caskin1 is known to be responsible for binding to CASK [3]. Similar functional binding motifs were identified in Mint1 and TIAM1, suggesting that Caskin1 may compete with these proteins for CASK binding in vivo [3].

Previously, we demonstrated that Abl interactor-2 (Abi-2) binds to the proline-rich, unstructured region of Caskin1 [2]. Besides CASK and Abi-2, our yeast two hybrid study identified several other proteins as potential interactors of Caskin1, including EphA2 (receptor tyrosine kinase), L1CAM (cell adhesion molecule), Myosin IB (cytoplasmic motor), Nck1 (adaptor), Neurexin 2 (neuronal cell adhesion molecule), Stathmin-like 3 protein (regulator of microtubule stability), Synaptotagmin (mediator of Ca^2+^-regulated vesicle fusion), Septin 4 (cell cycle regulator), and Siah1 (ubiquitin ligase) [2]. Additionally, a homolog of Caskin1 in Drosophila was found to be necessary for embryonic motor axon guidance by interacting with the leukocyte common antigen-related (Lar) receptor protein tyrosine phosphatase [4]. An SH2/SH3 adaptor protein, Dock (homolog of human Nck in Drosophila), was also identified as a binding partner of Caskin in the same work [4]. While Lar binds to the N-terminal SAM domain of Caskin, Dock has a different binding site, suggesting that they may form a tripartite complex in vivo. [4]. We have also demonstrated the binding of mammalian Nck to Caskin1 [5]. Moreover, we have shown that Nck recruits Caskin1 to EphB1, a receptor tyrosine kinase responsible for cell-cell contact dependent signalization [6]. In the complex, the SH2 domain of Nck binds to the activated receptor, while the SH3 domains of Nck bind the proline-rich C-terminal region of Caskin1 [5]. Intriguingly, a complex formation of the receptor, adaptor, and scaffold proteins results in tyrosine phosphorylation of Caskin1 on its SH3 domain.

Caskin1 has been associated with a number of pathological conditions. A study investigating transcriptomic and proteomic changes in a mouse model strain of autism spectrum disorders (ASDs) identified Caskin1 as a novel gene associated with the ASD-like phenotype [7]. Furthermore, down-regulation of the expression of Caskin1 by a microRNA (miR-21a-5p) has been found to promote the proliferation of porcine hemagglutinating encephalomyelitis virus [8]. Additionally, Caskin1 has been shown to be downregulated in an ischemia/reperfusion injury rat model system both at the mRNA and protein levels, indicating a potential role of Caskin1 in the pathomechanism of stroke [9]. It has also been shown that prenatal exposure to ethanol induces a significant decrease in the expression of Caskin1 in rats, indicating that maternal consumption of alcohol may affect Caskin-related synaptic functions in the brain [10]. Caskin1 may also be involved in the development of anaplastic large-cell lymphoma (ALCL) by being associated with nucleophosmin-anaplastic lymphoma kinase, a chimeric oncogene constitutively overexpressed in ALCL patients [11]. Moreover, it has been shown that the concentration of Caskin1 in the spinal dorsal horn increases during chronic pain and it contributes to various behavioral phenotypes, including nociception, gait, memory, and stress response in broad regions of the central nervous system [12]. More recently, a postsynaptic role of Caskins has been demonstrated in knockout mice with indications that they affect learning abilities by regulating spine morphology and AMPA receptor localization [13]. Additionally, the Caskin1 gene has been found to have deletions in some patients with TSC2/PKD1 contiguous gene deletion syndrome [14,15].

**Figure 1:**
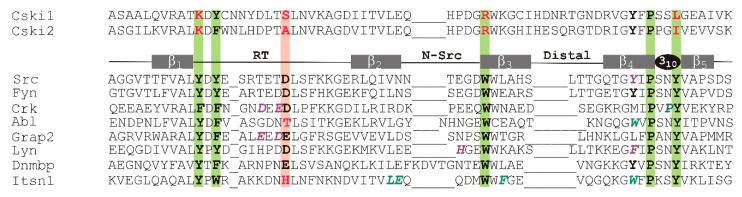
Sequence alignment of selected Src homology 3 (SH3) domains. The highly conserved residues that typically interact with the proline-rich ligand in many SH3 domains are shown in bold. Substitutions at some of the key conserved positions are indicated in red. The xP sites and the specificity sites are highlighted in green and pink backgrounds, respectively. Residues having an important role in ligand binding in specific SH3 domains are italicized and shown in aquamarine and magenta depending on whether they interact with the proline-rich segment or the flanking region of the ligand. The secondary structure elements are indicated above the sequence of c-Src. The SH3 sequences shown are as follows. Cski1: human Caskin1, residues 280–347, UniProt accession Q8WXD9; Cski2: human Caskin2, residues 280–347, Uniprot accession Q9WXE0, PDB 2KE9; Src: chicken c-Src, residues 80–142, UniProt accession P00523, PDB 1QWE; Fyn: human Fyn tyrosine kinase, residues 81–143, UniProt accession P06241, pDB: 4EIK; Crk: mouse c-Crk, N-terminal SH3 domain, residues 131–192, UniProt accession Q64010, PDB: 1CKA; Abl: mouse tyrosine-protein kinase, residues 60–121, UniProt accession P00520, PDB: 1ABO; Grap2: mouse GRB2-related adhesion protein 2, C-terminal SH3 domain, residues 262–322, UniProt accession O89100, PDB: 1OEB; Lyn: human tyrosine-kinase Lyn, residues 62–163, UniProt accession P07948, PDB: 1W1F; Dnmbp: human dynamin-binding protein C-terminal SH3 domain, residues 1512–1576, UniProt accession Q6XZF7, PDB: 4CC7; Itsn1: human Intersectin-1, residues 912–971, UniProt accession Q15811-1, PDB: 4IIM.

The SH3 domain of Caskins is atypical in a sense that, unlike in conventional SH3 domains [16,17,18], key conserved aromatic residues necessary for the recognition of canonical (PxxP) and non-canonical Pro-containing motifs in target signaling proteins are missing [19] (Figure 1). Accordingly, no Pro-rich interacting partners have been identified yet. Instead, as we have shown recently, Caskin1 SH3 selectively binds LPA in vitro [20], a signaling-born lysophospholipid mediator activating bona fide G-protein coupled receptors [21,22]. Specifically, as revealed by intrinsic tryptophan measurements, the SH3 domain of Caskin1 selectively binds to oleoyl (18:1) LPA, whereas no binding is observed for oleoyl LPC bearing a phosphocholine headgroup or for the related sphingolipid mediator sphingosine-1-phosphate [20]. Testing the effect of the saturation level of the hydrocarbon chain on binding further suggested that oleate esterified LPA or oleoyl-cyclic LPA is preferred over saturated palmitoyl LPA. Both fluorescence and ITC measurements have indicated a dependence of the binding interaction on the association state of LPA, with preferred binding of ~nM affinity to micellar LPA, suggesting that the SH3 domain has a preference for LPA-containing lipid surfaces compared to monomeric LPA. Chemical shift perturbation of ^15^N-HSQC spectra of Caskin1 SH3 domain indicates no major structural changes upon LPA addition, but reveals a discrete set of amino acids affected by LPA binding [20] (Figure S1). Mapping LPA-induced chemical shift changes to a homology model built based on the solution NMR structure of the SH3 domain of human Caskin2 has suggested that amino acids involved in LPA binding are likely to be distinct from the canonical proline-rich ligand binding groove in the SH3 domain of Src-kinase [20]. The goal of the present study was to obtain an atomic-level structure of human Caskin1 SH3 and analyze its structural features in comparison with SH3 domains known to bind canonical or non-canonical proline-rich peptides. The presented solution NMR structure provides structural evidence for a missing typical peptide binding groove seen in other SH3 domains as well as evidence that the binding surface for LPA is distinct from this altered peptide binding groove, supporting our hypothesis that the SH3 domain of Caskin1 has specialized for the binding of membrane surfaces with locally elevated LPA content during evolution.

## 2. Materials and Methods

### 2.1. Protein Expression and Purification

The coding sequence corresponding to the SH3 domain of human Caskin1 (Uniprot: Q8WXD9, residues: 284–346) was obtained from Integrated DNA Technologies as a synthetic gene construct and subsequently subcloned into a modified pET vector harboring a hexahistidine tag and a TEV protease recognition site by using NdeI and BamHI restriction sites. The 6x His-tagged protein was expressed in *E. coli* Rosetta pLysS cells. Cells were grown in 4 × 1000 mL LB medium and supplemented with ampicillin and chloramphenicol up to OD_600_ = 0.6 at 37 °C, while shaking at 250 rpm. Cells were harvested using centrifugation and resuspended in 1 L Minimal Media containing 1 g/L ^15^N-NH_4_Cl and 5 g/L ^13^C-d-glucose as single nitrogen and carbon sources, respectively. Protein expression was induced using 0.5 mM isopropyl-thio-ß-galactoside (Sigma-Aldrich) at 37 °C for 4 h. Cells were harvested using centrifugation for 20 min at 4 °C and then resuspended in lysis buffer (50 mM Tris-HCl, 300 mM NaCl, 0.5 M EDTA, 1 mM PMSF, 1× complete EDTA-free protease inhibitor cocktail (Roche), pH 8.0). After sonication (10 × 10 s), cell debris was removed using centrifugation (4 °C, 30 min) and the supernatant was purified using a Ni-NTA column (Macherey-Nagel, cat. no. 745400). The target protein was eluted with elution buffer (50 mM HEPES, 30 mM KCl, 5 mM TCEP, pH 7.5) containing 250 mM imidazole. The His-tag was cleaved using TEV protease while dialyzing against the lysis buffer. To remove the protease, residual undigested protein molecules, and digested His-tags, samples were loaded again onto a Ni-NTA column. The flow through was dialyzed against a buffer containing 20 mM K-phosphate, 100 mM KCl, 0.05% NaN_3_, 0.1 mM TCEP, pH = 7.2 (NMR buffer). Purity of the SH3 domain was monitored using SDS-PAGE.

### 2.2. NMR Spectroscopy

Multidimensional NMR experiments were carried out on a 600 MHz Varian NMR spectrometer equipped with a 5 mm indirect detection triple ^1^H^13^C^15^N resonance *z*-axis gradient probe in buffer containing 20 mM K-phosphate, 100 mM KCl, 0.05% NaN_3_, 0.1 mM TCEP, pH 7.2 at 10 °C. Protein concentration was ~0.6 mM. Backbone resonance assignment was obtained on the uniformly [U-^13^C,^15^N]-enriched SH3 domain of human Caskin1 using a combination of two-dimensional (2D) ^1^H-^15^N HSQC [23], three-dimensional (3D) gHNCACB [24], gCBACACONH [25], and gHBHA(CO)NH [26] experiments. Side-chain assignments involved 3D CC-TOCSY-NNH [27], 3D HCC-TOCSY-NNH [27], and 3D g-HCCH-TOCSY [28] measurements. Spectral processing, computer assisted spin-system analysis, and resonance assignment was carried out using Felix 2004 (Accelrys, Inc.). The ^1^H chemical shifts were referenced externally to 2,2-dimethylsilapentane-5-sulfonic acid (DSS), whereas ^13^C and ^15^N chemical shifts were referenced indirectly to DSS [29]. Position and length of secondary structure elements were initially determined from the deviations of Hα, Cα, and Cβ chemical shifts from random coil values using CSI [30]. Interproton distance restraints were obtained from aliphatic and aromatic 3D ^13^C-NOESY-HSQC [31], 3D ^15^N-NOESY-HSQC [32], 3D Met-Met-NOESY [33], and 2D ^1^H-^1^H NOESY spectra [34]. Structure calculations were performed with ARIA (Ambiguous Restraints for Iterative Assignment, version 2.2) [35] using a log-harmonic shape potential and Bayesian weighting for distance restraints [36]. In each of the seven iterations, the 50 lowest energy structures were used as templates for the next iteration and the 15 best structures were used for restraint violation analysis. The computational algorithm in the structure calculation employed torsional angle simulated annealing followed by torsional angle and Cartesian molecular dynamics cooling stages. Structural refinement was completed in a water shell. The stereochemical quality and structural statistics of the final ensemble were determined using PROCHECK [37], MolProbity [38], and the PDB validation server.

The ^15^N T_1_, T_2_ measurements [39,40] were collected on U-[^15^N]-enriched human Caskin1 at 20 °C at 14.1 T (corresponding to ^1^H Larmor frequencies of 600 MHz). The protein concentration was 0.2 mM. Backbone amide ^15^N T_1_ values were measured from two series of eight spectra (24 transients, interscan delay of 1.5 s) with the following relaxation delay times: T = 20, 100, 190, 290, 390, 530, 670, and 830 ms, and T = 20, 50, 100, 170, 240, 340, 480, and 710 ms. Amide ^15^N T_2_ values were obtained similarly: T = 10, 30, 50, 90, 130, 170, 210, and 250 ms, and T = 10, 30, 70, 90, 110, 150, 190, and 230 ms. To map the regions of backbone order and disorder, gradient- and sensitivity-enhanced ^15^N-HSQC experiments were used to collect amide proton saturation transfer data by recording spectra (32 transients) with and without water presaturation during a 5 s interscan delay [41]. Saturation transfer measurements were carried out in triplicates. Relaxation NMR data were analyzed with CCPNMR.

## 3. Results

### 3.1. 3D Solution Structure of the SH3 Domain of Human Caskin1

**Figure 2:**
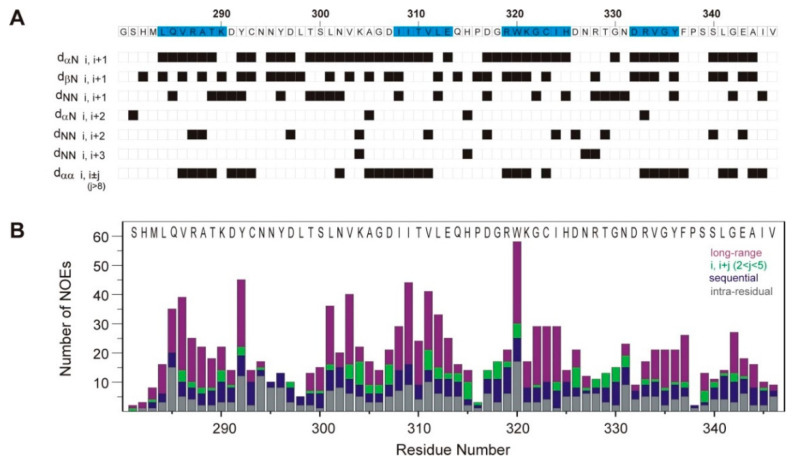
Summary of NMR structural parameters. (**A**) NOEs observed between residue pairs i and i + j are indicated in black. In the last row, NOEs between the alpha protons of residues separated by more than eight amino acids are diagnostic markers of strand-strand interactions in beta-sheets. The amino acid sequence of Caskin1 SH3 is shown at the top using the numbering of the complete human Caskin1 sequence. Residues with a consensus chemical shift index (CSI) indicating a beta-strand conformation as obtained from the analysis of the deviation of H_α_, C_α_, and C_β_ chemical shifts from random coil values are highlighted in blue. No 3_10_ helix is observed toward the C-terminus. (**B**) Distribution of NOE restraints. Intra-residue NOEs are in grey, sequential NOEs are in blue, NOEs between residues i and i + j, where 2 < j < 5 are in green, and long-range NOEs are in magenta.

**Figure 3:**
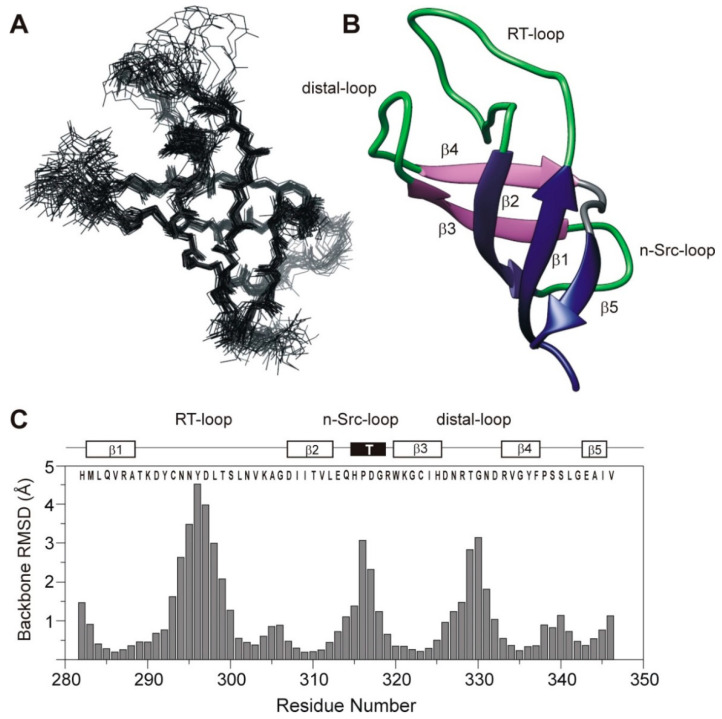
Results of NMR structure calculation. (**A**) Superposition of the 30 lowest-energy conformations obtained for the backbone atoms of the SH3 domain of human Caskin1. (**B**) Ribbon diagram of the most representative member of the lowest-energy structural ensemble. Secondary structure elements are labeled. Beta-strand β2 is shared by the front (β5, β1, β2) and back (β2, β3, β4) beta-sheets. (**C**) Average backbone RMSDs from the mean of the 30 lowest-energy conformations of the SH3 domain of human Caskin1. Secondary structure elements along the amino acid sequence are indicated at the top.

Sequence specific resonance assignments (Supplementary Table S1) and ^1^H-^1^H distance restraints for the human Caskin1 SH3 domain were obtained using standard 3D triple resonance experiments collected on uniformly ^13^C, ^15^N-enriched protein at 10 °C, as detailed in Materials and Methods. Elements of the secondary structure were initially identified via the analysis of the deviations of ^1^H_α_, ^13^C_α_, and ^13^C_β_ chemical shifts from random coil values and diagnostic inter-residue ^1^H-^1^H nuclear Overhauser effect (NOE) correlations (Figure 2A). As expected, beta-strands are the major elements of the secondary structure. This is evidenced by a consensus chemical shift index as well as stretches of strong sequential H_α,i_-HN_i+1_ NOEs complemented with long-range H_α,i_-H_α,i+j_ NOEs between the alpha protons of neighboring β-strands. Additionally, a large number of long-range NOEs were observed between the side chains of residues separated by more than five amino acids corresponding to strand-strand interactions. The distribution of sequential, medium, and long-range NOEs along the amino acid sequence is depicted in Figure 2B. NOE restraints used to determine the tertiary structure of the domain together with statistics for the lowest-energy structural ensemble (Figure 3A) are summarized in Table 1. In ordered protein regions, over 80% of the non-proline, non-glycine residues were in the most favored regions of the Ramachandran plot. The exceptions included residues in more ordered regions of the RT-loop (D291, Y292, S300, N302, K304) and in the n-Src-loop (Q314) assuming (Φ, Ψ) angles in the additionally allowed regions of the Ramachandran plot. A representative element of the lowest-energy structural ensemble is depicted in Figure 3B. Accordingly, the Caskin1 SH3 domain displays a tertiary fold characteristic of SH3 beta-sandwiches. Specifically, a hydrophobic core is defined by two orthogonal antiparallel beta-sheets, with one of the strands (β2) shared by the two sheets. The hydrophobic core is stabilized by van der Waals interactions between bulky hydrophobic side chains of β2 (I309, V311, L312) and β3 (W320, I324), with contribution from β1 (L284, V286) and the RT-loop (L301). Between β2 and β3, as part of the n-Src-loop, residues ^315^HPDG^318^ form a type I turn stabilized by multiple i, i + 3 (between H315 and G318) and i, i + 4 (between H315 and R319) backbone H-bonds. Unlike in many other SH3 domains [42], no presence of a 3_10_ helix is observed toward the C-terminus. While residues in the beta-strands show a high degree of convergence (with overall RMSDs from the mean structure of 0.8 and 1.3 Å for backbone and all heavy atoms, respectively), most of the loop regions display a high degree of flexibility (Figure 3C). However, besides the network of backbone H-bonds stabilizing the overall fold, a number of side chain interactions occur between more peripheral elements restricting the motional freedom of these segments. This includes a salt bridge between the side chains of R287 and E343 stabilizing the contact between β1 and β5. It is noteworthy that the distal-loop (D326-R333) is involved in multiple H-bond interactions with β2 (between the side chains of D307 and N327) and the RT-loop (between the side chains of N302 and R333). The distal-loop is further stabilized by favorable electrostatic interactions between the side chains of D326 and K304 (RT-loop). Additionally, an H-bond between the side chains of D326 and N331 occurs in about half of the lowest-energy structural ensemble restricting the conformational space of the loop. Backbone-side chain contacts are also prevalent including the side chains of D307 (β2), D326 (distal-loop), and R333 (distal-loop). Importantly, the beginning of the RT-loop is restricted by two backbone H-bonds (K290-A305 and Y292-V303) extending the tie between β1 and β2.

### 3.2. Solvent Exchange and Backbone ^15^N Relaxation

**Figure 4:**
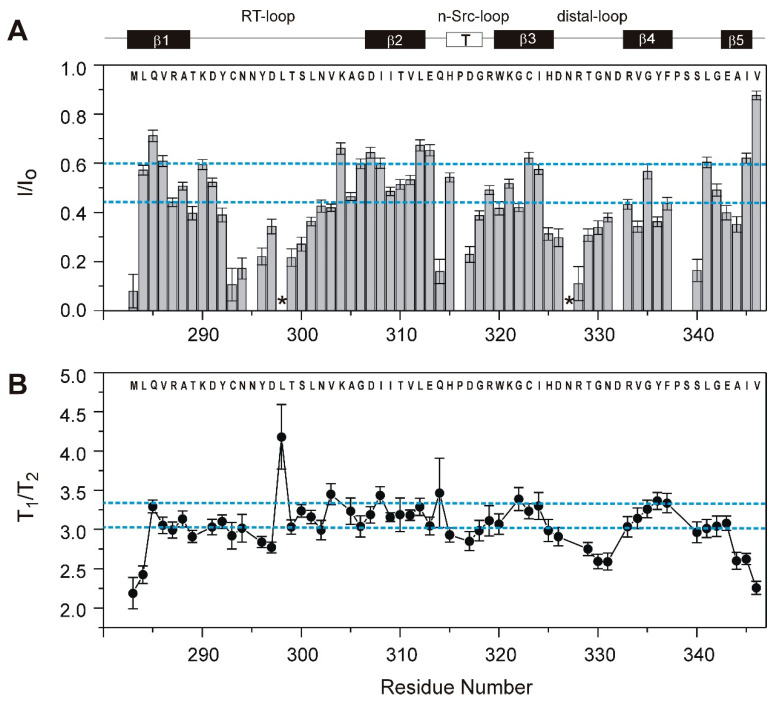
Summary of NMR parameters diagnostic of slow motions and local unfolding events in the SH3 domain of human Caskin1. (**A**) Ratio of relative peak intensities with (I) and without (I_o_) presaturation of the solvent resonance and (**B**) ratio of the longitudinal (T_1_) and transverse (T_2_) relaxation times as a function of the amino acid sequence. Error bars are shown. Dashed lines correspond to the mean and the mean plus one standard deviation. At residues marked with an asterisk in (**A**) fast solvent exchange obscures the analysis. Secondary structural elements are indicated at the top. Longitudinal and transverse relaxation times averaged around T_1_ = 449 ± 42 ms and T_2_ = 149 ± 23 ms, respectively, matching the values expected for an ~70-residue globular protein at the investigated temperature (20 °C).

Hydrogen exchange rates characteristic of backbone hydrogen bonding interactions and local unfolding events in proteins [43,44] in general follow the distribution of secondary structure elements in Caskin1 SH3. This is shown in Figure 4A, where the relative intensity (I/I_o_) of ^1^H-^15^N correlations collected with and without solvent presaturation is depicted along the amino acid sequence. The two longest continuous segments exhibiting rapid hydrogen exchange are C293-S300 of the RT-loop and H325-G330 of the distal-loop. The most protected surface from solvent exchange is comprised of β2 and the middle segments of β1 and β3. β4 is considerably more susceptible to solvent exchange, whereas via two strong H-bonds to Q285 of β1, the C-terminal half of β5 gains an increased protection.

In some SH3 domains, such as in Fyn tyrosine kinase, NMR measurements have indicated the presence of a low-populating folding intermediate in equilibrium with its unfolded and fully folded states at ambient temperatures [45]. As low populated higher energy states including partially unfolded states could play a role in molecular recognition processes mediated by the SH3 domain of Caskin1 as well, we examined the possibility of the contribution of conformational exchange to transverse relaxation. The ratio of longitudinal (T_1_) and transverse (T_2_) ^15^N relaxation times, a diagnostic marker of conformational exchange processes on the μs-ms timescale, is plotted in Figure 4B as a function of the amino acid sequence. Values of T_1_/T_2_ exceeding the average by more than one standard deviation have been found sporadically throughout the protein at residues L298 (RT), V303 (RT), I308 (β2), Q314 (n-Src), G322 (β3), and Y328 (β4). Among them, L298, Q314, and Y328 exhibit intensive solvent exchange as well. The absence of continuous segments of elevated T_1_/T_2_ values shows that slow conformational fluctuations do not provide a significant contribution to transverse relaxation and the Caskin1 SH3 domain is fairly rigid on the μs-ms timescale.

### 3.3. Mapping LPA-Induced Chemical Shift Perturbations on the NMR Structure of Human Caskin1 SH3 Domain

**Figure 5:**
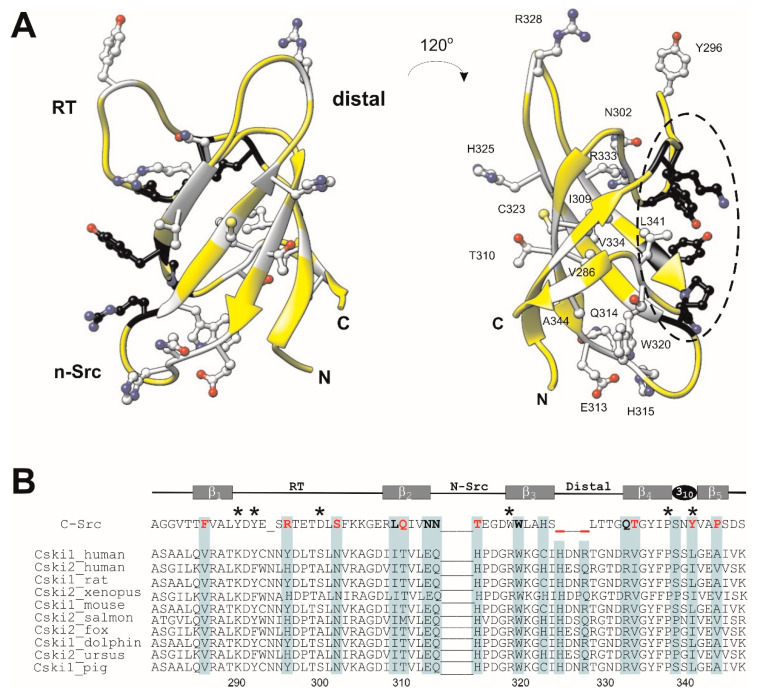
Interaction of human Caskin1 SH3 with oleoyl LPA. (**A**) Representative element of the lowest-energy structural ensemble of the SH3 domain of human Caskin1 highlighting the residues with above average oleoyl LPA-induced (lipid-to-protein molar ratio of 10:1) backbone (^1^H, ^15^N) chemical shift change (Figure S1). The region corresponding to the peptide binding groove in the SH3 domain of Src kinase are shown in black. (**B**) Sequence alignment of selected Caskin1 and Caskin2 SH3 domains. Residues with chemical shift change exceeding the average upon the addition of LPA are highlighted in blue revealing a high degree of conversion in Caskins. The sequence of the SH3 domain of c-Src is shown at the top for comparison together with the secondary structure elements. Non-conserved amino acid replacements affected by LPA binding is indicated in red. Amino acid positions corresponding to the peptide binding groove in c-Src are marked by an asterisk. The SH3 sequences shown as follows. Src: c-Src, *Gallus gallus* (UniProt P00523); Caskin1, *Homo sapiens* (UniProt Q8WXD9); Caskin2, *Homo sapiens* (Uniprot Q9WXE0); Caskin1, *Rattus norvegicus* (UniProt Q8VHK2); Caskin2, *Xenopus laevis* (Uniprot Q6DD51); Caskin1, *Mus musculus* (UniProt Q6P9K8); Caskin2, *Salmo salar* (UniProt A0A1S3QUC9); Caskin2, *Vulpes vulpes* (UniProt A0A3Q7T5U3); Caskin1, *Lipotes vexillifer* (UniProt A0A340X375); Caskin2, *Ursus arctos horribilis* (UniProt A0A3Q7TV80); Caskin1, *Sus scrofa* (UniProt I3LCF2).

As we have shown previously, addition of oleoyl LPA to the human Caskin1 SH3 domain (lipid-to-protein molar ratio of 10) induces above-average chemical shift perturbations (Figure S1) in a discrete set of amino acids involving residues of β1–β5 (V286, I309-T310, C323, H325, R328, R333-V334, A344), the ^313^EQH^315^ triad of the n-Src-loop together with W320 at the beginning of β3, and sporadically at other positions, primarily with side chains capable of H-bond formation (Y296, N302, S339). Notably, the binding of LPA induces a peak doubling for a small number of residues including V346 and I308 as well as the side chain NH_2_ groups of N302 and N327. The affected amino acid positions are mapped onto the solution NMR structure of the human Caskin1 SH3 domain in Figure 5A. The majority of the perturbations in the hydrophobic core of the domain suggest that besides the favorable electrostatic interaction of arginines (and depending on their charged state, perhaps the histidines as well) with the LPA headgroups, the hydrophobic acyl chains of LPA may insert deeply into the protein interior. The involvement of residues with H-bond donor/acceptor side chains also suggests the rearrangement of bound water molecules upon LPA binding. Importantly, the most significant changes in the chemical environment upon the interaction with LPA are detected in regions distinct from the canonical PxxP or non-canonical proline-rich peptide binding site of SH3 domains in general (cf below). Based on the Gonnet Pam250 matrix [46], using the groupings of ‘STA’, ‘NEQK’. ‘NHQK’, ‘NDEQ’, ‘QHRK’, ‘MILV’, ‘MILF’, ‘HY’, ‘FYW’, and ‘C’ for the analysis of sequence similarity, the majority of LPA-affected residues are well conserved among Caskins (Figure 5B), whereas they are not conserved among SH3 domains in general, indicating that LPA recognition is likely to be specific to Caskins.

### 3.4. Comparison with the SH3 Domain of Human Caskin2

**Figure 6:**
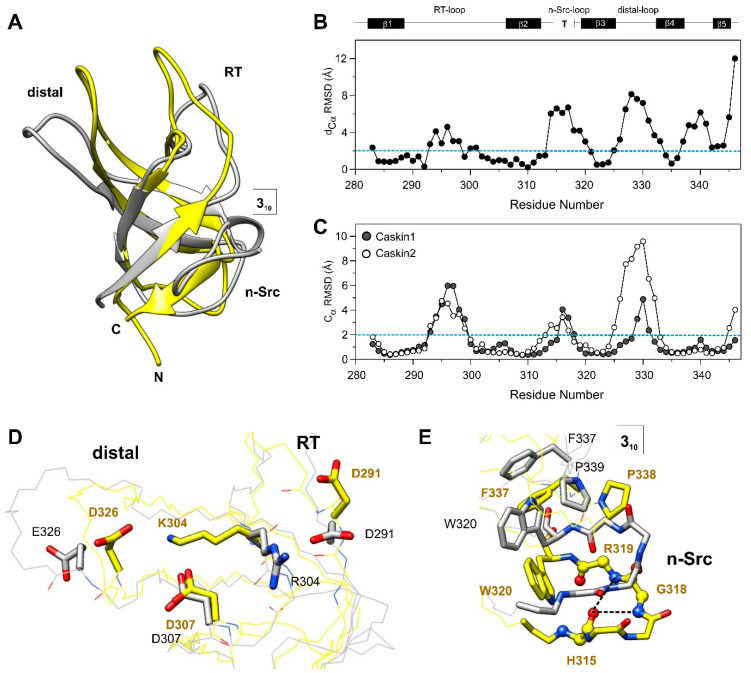
Structural comparison of the SH3 domains of human Caskin1 and Caskin2. (**A**) Superimposed ribbon diagrams of the lowest-energy element of the structural ensemble of the SH3 domains of human Caskin1 (yellow, PDB: 7ATY) and Caskin2 (grey, PDB: 2KE9 [18]). (**B**) C_α_ positional differences between the mean structures of the Caskin1 and Caskin2 SH3 structural ensemble. (**C**) Average C_α_ RMSDs based on pairwise distances between matched atoms in the lowest-energy structural ensemble of the SH3 domain of human Caskin1 (grey) and Caskin2 (white). Dashed lines in (**B**) and (**C**) at 2 Å are for better viewing. (**D**,**E**) Superimposed diagrams of the Caskin1 (yellow) and Caskin2 (grey) SH3 domains highlighting (**D**) stabilizing electrostatic interactions between the RT- (K304) and the distal-loops (D326) and (**E**) the formation of a type I turn in the n-Src-loop of Caskin1. Stabilizing H-bonds between the backbone atoms of H315, G318, and R319 are indicated by dashed lines. Hydrophobic interactions of W320 with F337 and P339 of the 3_10_-helical region in Caskin2 SH3 are shown in grey. Nitrogen and oxygen atoms are shown in blue and red, respectively. See text for details.

Comparison of the Caskin1 SH3 domain and its isoform, human Caskin2 (PDB: 2ke9 [18]) reveals differences primarily in the loop regions as well as in the linker connecting the β4 and β5 beta-strands. This is shown in Figure 6A, where the lowest-energy element of the structural ensemble of Caskin1 and Caskin2 is superimposed, and in Figure 6B, where C_α_ positional differences (d_Cα_) between the mean structures of the two ensembles are depicted along the amino acid sequence. The region exhibiting the largest difference between the two isoforms is the distal- and the n-Src-loop with values of d_Cα_ exceeding 6 Å. Among the two, the distal-loop shows a significantly larger conformational heterogeneity in Caskin2 with C_α_ RMSDs of the structural ensemble approaching 10 Å in the central segment of the loop (Figure 6C). In Caskin1, the H325-N331 region appears to be more defined due to favorable electrostatic interactions between the side chains of D326 and K304 of the RT-loop (Figure 6D). The NH_3_^+^ group of the lysine is further stabilized by the proximate D307. In Caskin2, the lysine is replaced by an arginine, and instead of the distal-loop, it forms contacts with D291 located in the N-terminal end of the RT-loop. Another important difference between the two isoforms is the n-Src-loop, which in Caskin1 assumes a type I turn stabilized by two H-bonds (Figure 6E). In Caskin2, W320 (β3), following immediately the n-Src-loop, forms hydrophobic contacts with F337 of β4 and P339 of the 3_10_ helix, pulling β3 slightly away from β2, yielding a looser n-Src-loop and hindering the formation of a well-defined turn. While in Caskin2 the 3_10_ helix (^339^PGI^341^) is stabilized by two hydrogen-bonds, in Caskin1 the corresponding ^338^PSSL^341^ segment appears to be more flexible. The lack of the formation of a stable 3_10_ helix in Caskin1 is suggested by the analysis of chemical shifts, the observed strong solvent exchange at S340 (Figure 4A), and the low number of NOEs in the region. We note that despite the high degree of overall sequence similarity between the SH3 domains of Caskin1 and Caskin2, this region, i.e., the linker between β4 and β5 together with β5, shows significant dissimilarities (Figure 1). It is noteworthy that the arrangement of the residues in the altered peptide binding groove in the SH3 domain of Caskin1 and Caskin2 are highly similar. Among the regions found to be affected by LPA in Caskin1 SH3, the n-Src-loop differs the most.

### 3.5. Comparison with other SH3 Domains

**Figure 7:**
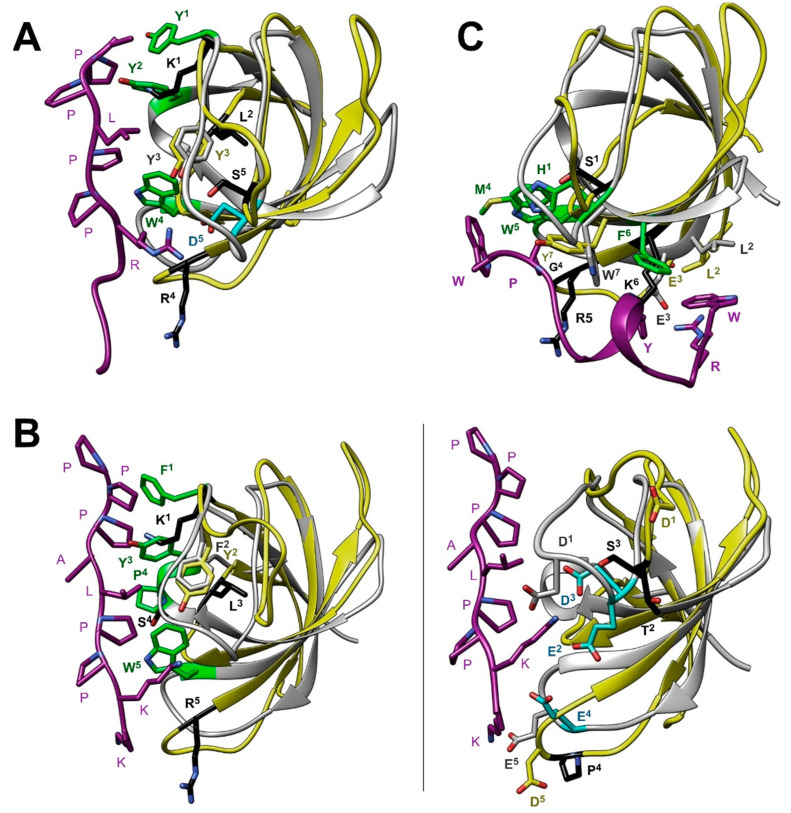
Superimposed ribbon diagrams of the SH3 domains of human Caskin1 (yellow, PDB: 7ATY) and (**A**) c-Src complexed with a proline-rich ligand (grey, PDB: 1QWE [47]), (**B**) c-Crk complexed with a proline-rich ligand (grey, PDB: 1CKA [48]), (**C**) intersectin-1 complexed with a synthetic proline-rich peptide (grey, PDB: 4IIM [49]). Side chains that have been found crucial for ligand binding in typical SH3 domains are indicated as sticks. For clarity, in the case of c-Crk, the PxxP (**left**) and specificity (**right**) site are shown separately. Aromatic (green) and acidic (cyan) residues, which have a key role in binding and are replaced by other residues (black) in Caskin1 are highlighted. Residue numbers are omitted for clarity. Superscripts are used to mark the same amino acid position in the superimposed structures to highlight the replacements in Caskin1. Oxigen, nitrogen, and sulfur atoms are in red, blue, and yellow, respectively. Ligands are shown in magenta.

As we noted in the introduction, the SH3 domains of human Caskin1 and Caskin2 are unique in the sense that some of the aromatic residues having a major role in the binding of Pro-rich sequences in other SH3 domains are substituted by amino acids with positively charged or small hydrophobic side chains. This is demonstrated in Figure 7A, where human Caskin1 is superimposed on the complex of Src SH3 with APP12 (^1^APPLPPRNRPRL^12^), a canonical high-affinity Pro-rich peptide selected from a phage display library [47]. The two dipeptide units of APP12 (Ala_1_-Pro_2_ and Leu_4_-Pro_5_) bind to the hydrophobic clefts formed by Y90 (in Figure 7A, Y^1^), Y136 (Y^2^) (pocket 1) and Y92 (Y^3^), W118 (W^4^), and Y136 (Y^2^) (pocket 2) of Src-SH3, respectively. Among the flanking residues, Arg_7_ packs against the side chain of W118 and forms a salt bridge with D99 (D^5^) near the RT-loop (pocket 3). As shown in Figure 7A, in Caskin1, three of the key aromatic residues are substituted with K290 (K^1^), R319 (R^4^), and L341 (L^2^), whereas the aspartate of the specificity pocket is replaced by a serine.

Besides the xP binding site, even larger differences exist between the specificity site of the SH3 domain of Caskin1 and that of c-Crk (Figure 7B). As revealed by the X-ray structure of the N-terminal Crk-SH3 domain in a complex with a high affinity peptide (^1^PPPALPPKKR^10^) from the guanine nucleotide exchange factor (C3G) [48], a lysine residue is tightly coordinated by three acidic residues of the RT-loop. Binding of the flanking region is stabilized by three simultaneous hydrogen-bonds between the carboxylates and the sp3 hybridized amino group of the lysine. As noted by the authors, the high specificity of the interaction is highlighted by the observed disorder in the complex with a mutant peptide, where Lys_8_ is replaced by an arginine. While the lysine-carboxylate interaction is unique to c-Crk SH3 and its relatives, it is a remarkable example of a specifically evolved mode of recognition to overcome the limited variability of binding motifs on the proline-rich ligand. When comparing it to Caskin1 SH3, in addition to the missing hydrophobic interaction at the canonical binding site (Figure 7B, left), there is significantly less acidic residues in the RT-loop and the ones present are positioned in a non-optimal geometry for the binding of positively charged (either Arg or Lys) flanking residues in Pro-rich peptides (Figure 7B, right).

The adaptability and the divergent strategies of recognition by SH3 domains are further exemplified by a complex of one of the SH3 domains of human intersectin-1 (Itsn1) with a non-canonical peptide ligand [49]. A synthetic peptide ^1^WRDSSGYVMGPW^12^ with a K_d_ of ~50 μM has been shown to interact with a specificity site and a novel exosite on the surface of the Itsn1 SH3 domain, different from the canonical PxxP binding site. However, even the interaction with the specificity site possesses features differing from common motifs observed in SH3 domains. Specifically, instead of positively charged flanking residues, the C-terminal GPW triad of the ligand packs against a hydrophobic pocket encompassed by M948 (M^4^), W949 (W^5^), and W959 (W^7^) (Figure 7C). The longer WRDSSGYVM region of the peptide binds to an exosite, where hydrophobic interactions together with hydrogen-bonds and a salt bridge stabilize the complex.

## 4. Discussion

SH3 domains are one of the most prevalent protein modules in nature involved in a variety of cellular functions including cell growth and differentiation, cytoskeletal rearrangements and cell motility, signal transduction, protein degradation, and immune response [19,50]. Primarily, they are known to recognize Pro-rich motifs, in particular the canonical PxxP motifs in proteins [16,51], forming a left-handed, polyproline type II helix in either of two well-defined orientations (class I and II) [47,52]. However, in recent years, there is growing evidence of alternative binding motifs underlying the broader range of specificity of SH3 domains. These atypical binding motifs include both proline-containing motifs (e.g., PxxxPR [53], PxxDY [54]) and those spared of proline, e.g., RxxK [55,56,57] and RKxxY [58,59]. In the latter, the phosphorylation state of the tyrosine has been shown to regulate the binding interaction suggesting that peptide recognition by SH3 may be coupled to phosphotyrosine signaling. This is supported by subsequent studies showing also that phosphorylation of tyrosines in SH3 domains themselves can lead to the inhibition of partner binding. Specifically, in a recent study of Abl1 and Abl2 SH3 domains, we have shown that two specific simultaneously-phosphorylated tyrosines hinder ligand binding by sterically blocking the ligand binding groove with the phosphate groups [60].

In both canonical and non-canonical peptide recognition, besides the set of conserved, primarily aromatic residues comprising the binding groove, an additional key element of interaction is the specificity site contributed mainly by the RT- and n-Src-loops. In most cases, it involves a pocket of negatively charged residues recognizing a basic Arg or Lys on the binding partner flanking the central motif (Figure 7A,B). Additionally, a conserved tryptophan following the n-Src-loop (Figure 1) often forms stabilizing van der Waals and cation-π interactions with the binding partner. Importantly, the length and sequence of the RT- and n-Src-loops display a variety among SH3 domains resulting in structurally diverse specificity sites, which may recognize peptide motifs other than the canonical with a positively charged flanking residue (Figure 7C) [49]. Additionally, besides the utilization of specific recognition elements, SH3 domains have also been shown to associate with other proteins via tertiary contacts without the involvement of a specific binding motif. Examples include the interaction between the Sla1 SH3 domain and ubiquitin [61] as well as the Fyn SH3 domain and the SAP SH2 domain [62]. Taken together, despite the common architecture of the SH3 domain, via a combinatorial use of the xP binding site and the specificity site, diverse strategies for partner recognition are observed across the family [17].

The SH3 domain of human Caskin1 differs from typical SH3 domains as some of the key aromatic residues involved in ligand binding are substituted by basic or small hydrophobic residues (Figure 1 and Figure 7A,B). Additionally, some of the negatively charged residues in the RT-loop are also missing. Instead, the distal-loop possesses an acidic patch involving D326 and D332 (unlike for instance the SH3 domain of Src kinase). Additionally, a repositioning of acidic residues is found in the n-Src-loop. Mapping of the residues involved in LPA binding to the NMR structure of human Caskin1 SH3 presented for the first time in the current study provides structural evidence for the missing proline-rich peptide binding groove and undoubtedly shows that LPA binding involves a protein region distinct from the peptide binding groove present in SH3 domains in general. The SH3 domain of human Caskin1 is not the only SH3 domain reported to date with a capability of lipid binding. Helical extended specialized SH3 domains have been reported to bind acidic phospholipids [63,64]. More recently, lipid binding by c-Src SH3 has been shown involving residues of the RT- and n-Src-loops forming a binding site opposite to the classical peptide binding site but overlapping with the region interacting with the Unique domain of c-Src. This suggests a complex interplay with membranes and an additional layer of regulation in c-Src signal transduction [65].

In conclusion, while adaptability is essential for the evolution of novel pathways and the modulation of signaling events, discrimination between potential binding partners is of high importance. We hypothesize that selective binding to LPA allows the anchoring of Caskin1 SH3 to membrane microdomains where LPA accumulates due to elevated production in specific signaling events. Thus, by serving as docking sites for the SH3 domain, lysophosphatidate residing within the plasma membrane may contribute to the guiding of Caskin1 interaction networks. For instance, based on the competing binding of Mint1- and Caskin1-CASK complexes to the cytoplasmic tail of neurexin, one can hypothesize that a coincident LPA signaling event might modulate the signaling of this protein network. Taken together, our structural data support a novel mode of recognition in Caskin1 signal transduction, where affinity and specificity can be tuned by imposing a spatial and temporal regulation of its interaction domain by LPA.

## Figures and Tables

**Table 1:** Statistics and stereochemical quality of the lowest-energy NMR structural ensemble (30 structures) of the human Caskin1 SH3 domain in aqueous buffer (20 mM K-phosphate, 100 mM KCl, 0.05% NaN_3_, 0.1 mM TCEP, pH 7.2) at 10 °C. The analysis was carried out using Procheck and the Protein Structure Validation Suite (PSVS). Glycines, prolines, and residues in disordered regions were excluded from the Ramachandran analysis.

Distance restraints from NOEs	
unambiguous	809
intraresidue	307
sequential	152
i–i + j, where j = 2, 3, or 4	74
i–i + j, where j > 4	276
ambiguous	180
Ensemble RMSD values	
All backbone atoms	1.3 Å
All heavy atoms	1.8 Å
All backbone atoms in ordered regions	0.8 Å
All heavy atoms in ordered regions	1.3 Å
Statistics	
Ramachandran plot statistics (ordered protein regions)	
Residues in most favored regions [A, B, L], %	81.5
Residues in additionally allowed regions [a, b, l, p], %	18.3
Residues in generously allowed regions [~a, ~b, ~l, ~p], %	0.2
Residues in disallowed regions, %	0.0
Main-chain statistics	
SD of ω angle, degrees	3.9
Bad contacts/100 residues	0
C_α_ chirality, SD of ζ angle, degrees	1.2
SD of H-bond energy, kcal/mol	0.9
Overall G-factor	−0.1
Side-chain statistics	
χ-1 gauche minus SD, degrees	7.3
χ-1 trans SD, degrees	9.1
χ-1 gauche plus SD, degrees	9.7
χ-1 pooled SD, degrees	10.7
χ-2 trans SD, degrees	12.0

## Data Availability

The data presented in this study are openly available in the RCSB PDB database (accession number 7ATY) and in the Biological Magnetic Resonance Bank (accession number BMRB 50543).

## Supplementary Materials

The following are available online at https://www.mdpi.com/2073-4409/10/1/173/s1, Figure S1: Oleoyl LPA-induced NMR spectral changes. Table S1: Assigned chemical shifts of the SH3 domain of human Caskin1.
