# Supplementary material for: Solution NMR Structure of the SH3 Domain of Human Caskin1 Validates the Lack of a Typical Peptide Binding Groove and Supports a Role in Lipid Mediator Binding
Source: Cells. 2021 Jan 16;10(1):173. doi: 10.3390/cells10010173 (PMC7830187; doi:10.3390/cells10010173)
Supplement: Supplementary file 1 [file cells-10-00173-s001.zip › caskin1-sh3-supplementary-201214/caskin1-sh3-suppl-info.pdf]

SUPPLEMENTARY INFORMATION

Solution NMR structure of the SH3 domain of human Caskin1 validates the lack of a typical peptide binding groove and supports a role in lipid mediator binding

Orsolya Tőke[1*], Kitti Koprivanacz[2], László Radnai[2,†], Balázs Merő[2], Tünde Juhász[2], Károly Liliom[3], László Buday[2*]

[1]*Laboratory for NMR Spectroscopy, Research Centre for Natural Sciences, 2 Magyar tudósok körútja, H-1117 Budapest, Hungary*

[2]*Institute of Enzymology, Research Centre for Natural Sciences, 2 Magyar tudósok körútja, H-1117 Budapest, Hungary*

[3]*Department of Biophysics and Radiation Biology, Semmelweis University, 37-47 Tűzoltó utca, H-1094 Budapest, Hungary*

[†]*Current Address: Department of Molecular Medicine and Department of Neuroscience, The Scripps Research Institute, 130 Scripps Way, Jupiter, FL 33458, USA*

Corresponding authors:

Orsolya Toke, Ph.D.
Laboratory for NMR Spectroscopy
Research Centre for Natural Sciences
2 Magyar tudósok körútja
H-1117 Budapest, HUNGARY

phone: +36-1-382-6575
email: toke.orsolya@ttk.hu

and

László Buday, M.D., Ph.D., member of HAS
Institute of Enzymology
Research Centre for Natural Sciences
2 Magyar tudósok körútja
H-1117 Budapest, HUNGARY

phone: +36-1-382-6700
email: buday.laszlo@ttk.hu

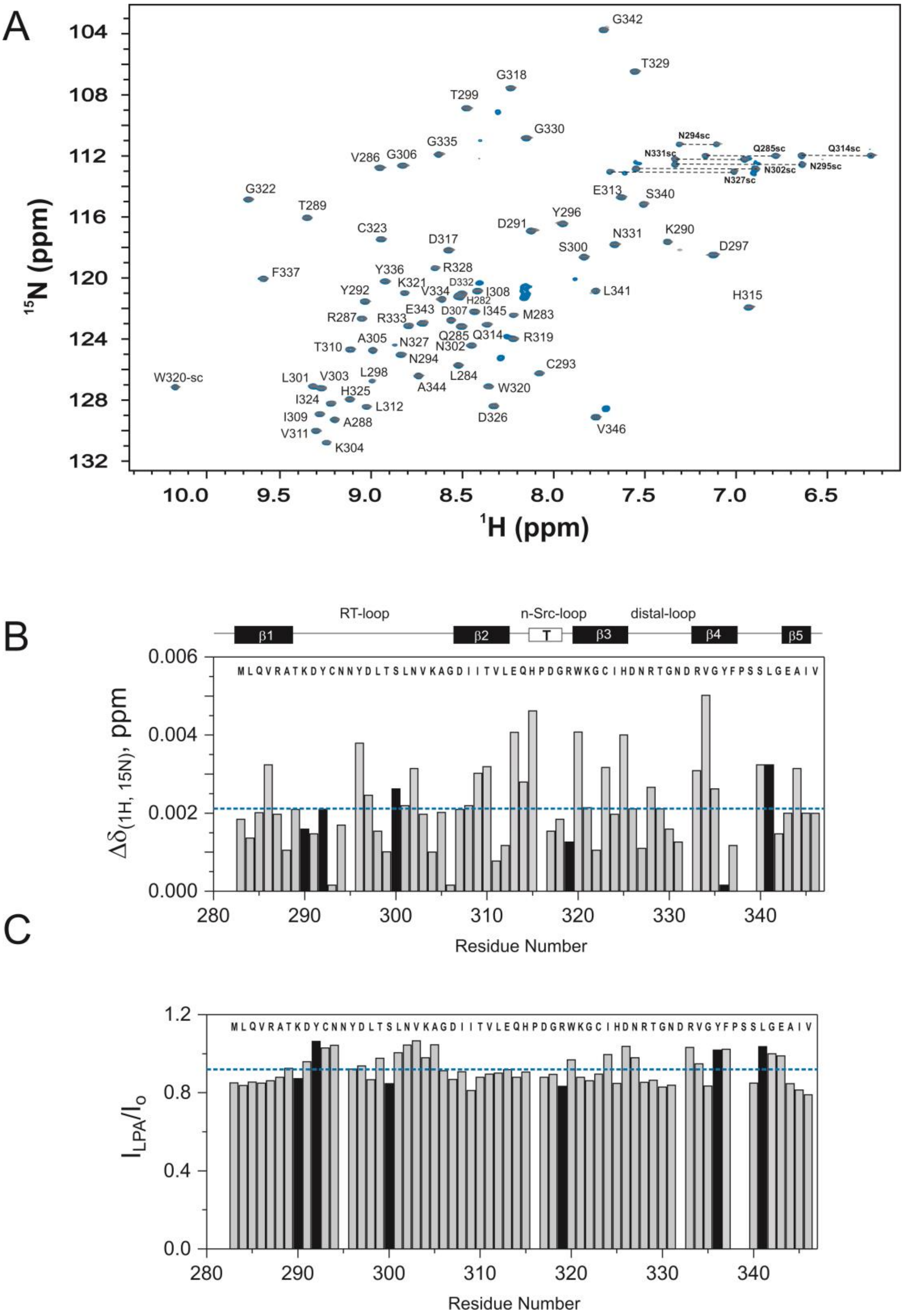

Figure S1. Oleoyl LPA-induced NMR spectral changes. (A) Superposition of ^{1}H-^{15}N HSQC spectra (600 MHz for ^{1}H, 20 °C) of uniformly ^{15}N-enriched SH3 domain of human Caskin1 in the absence (grey) and in the presence (blue) of oleoyl LPA at a lipid-to-molar ratio of 10. (B) Combined (^{1}HN, ^{15}N) chemical shift perturbations induced by LPA. Secondary structure elements are indicated at the top. (C) Intensity changes of backbone amide resonances upon the addition LPA. Key residues corresponding to the peptide binding groove in the SH3 domain of Src kinase are shown at the top in both (B) and (C). Dashed lines correspond to the mean value.

Table S1. Assigned chemical shifts for the SH3 domain of human Caskin1 (20 mM K-phosphate, 100 mM KCl, 0.05% NaN_3, 0.1 mM TCEP, pH 7.2 at 10°C).

Residue number	Residue name	Atom name	Chemical shift (ppm)	Chemical shift error (ppm)	Ambiguity index*
281	SER	HA	5.35	0.01	1
281	SER	HB2	3.81	0.01	1
281	SER	HB3	3.81	0.01	1
281	SER	CA	58.3	0.1	1
281	SER	CB	63.8	0.1	1
282	HIS	H	8.51	0.01	1
282	HIS	HA	4.64	0.01	1
282	HIS	HB2	3.08	0.01	1
282	HIS	HB3	3.08	0.01	1
282	HIS	CA	56.2	0.1	1
282	HIS	CB	30.6	0.1	1
282	HIS	N	121.1	0.1	1
283	MET	H	8.23	0.01	1
283	MET	HA	4.40	0.01	1
283	MET	HB2	1.83	0.01	1
283	MET	HB3	1.83	0.01	1
283	MET	HG2	2.42	0.01	1
283	MET	HG3	2.42	0.01	1
283	MET	CA	55.3	0.1	1
283	MET	CB	33.4	0.1	1
283	MET	N	122.0	0.1	1
284	LEU	H	8.53	0.01	1
284	LEU	HA	4.39	0.01	1
284	LEU	HB2	1.64	0.01	2
284	LEU	HB3	1.00	0.01	2
284	LEU	HD11	0.93	0.01	2
284	LEU	HD12	0.93	0.01	2
284	LEU	HD13	0.93	0.01	2
284	LEU	HD21	0.81	0.01	2
284	LEU	HD22	0.81	0.01	2
284	LEU	HD23	0.81	0.01	2
284	LEU	CA	55.8	0.1	1
284	LEU	CD1	21.2	0.1	1
284	LEU	CD2	26.1	0.1	1
284	LEU	N	125.2	0.1	1
285	GLN	H	8.52	0.01	1
285	GLN	HA	5.60	0.01	1
285	GLN	HB2	1.92	0.01	2
285	GLN	HB3	1.66	0.01	2
285	GLN	HG2	2.29	0.01	1
285	GLN	HG3	2.29	0.01	1
285	GLN	HE21	7.18	0.01	2

*Ambiguity index values are defined as in the Biological Magnetic Resonance Data Bank. 1: unique; 2: ambiguity of geminal atoms or geminal methyl proton groups; 3: aromatic atoms on opposite sides of symmetrical rings; 4: intraresidue ambiguity.

Table S1 Continuation. Assigned chemical shifts for the SH3 domain of human Caskin1 (20 mM K-phosphate, 100 mM KCl, 0.05% NaN_3, 0.1 mM TCEP, pH 7.2 at 10°C).

Residue number	Residue name	Atom name	Chemical shift (ppm)	Chemical shift error (ppm)	Ambiguity index*
285	GLN	CB	33.1	0.1	1
285	GLN	CG	38.2	0.1	1
285	GLN	N	122.5	0.1	1
285	GLN	NE2	111.5	0.1	1
286	VAL	H	8.97	0.01	1
286	VAL	HA	4.97	0.01	1
286	VAL	HB	1.84	0.01	1
286	VAL	HG11	0.88	0.01	1
286	VAL	HG12	0.88	0.01	1
286	VAL	HG13	0.88	0.01	1
286	VAL	HG21	0.88	0.01	1
286	VAL	HG22	0.88	0.01	1
286	VAL	HG23	0.88	0.01	1
286	VAL	CG1	20.2	0.1	1
286	VAL	CG2	20.2	0.1	1
286	VAL	CA	58.3	0.1	1
286	VAL	CB	35.5	0.1	1
286	VAL	N	112.0	0.1	1
287	ARG	H	9.06	0.01	1
287	ARG	HA	5.13	0.01	1
287	ARG	HB2	1.77	0.01	2
287	ARG	HB3	1.83	0.01	2
287	ARG	HG2	1.39	0.01	2
287	ARG	HG3	1.50	0.01	2
287	ARG	HD2	3.21	0.01	2
287	ARG	HD3	3.26	0.01	2
287	ARG	CA	53.5	0.1	1
287	ARG	CB	32.5	0.1	1
287	ARG	CG	29.4	0.1	1
287	ARG	CD	43.4	0.1	1
287	ARG	N	122.1	0.1	1
288	ALA	H	9.21	0.01	1
288	ALA	HA	4.52	0.01	1
288	ALA	HB1	1.41	0.01	1
288	ALA	HB2	1.41	0.01	1
288	ALA	HB3	1.41	0.01	1
288	ALA	CA	52.3	0.1	1
288	ALA	CB	20.4	0.1	1
288	ALA	N	128.7	0.1	1
289	THR	H	9.36	0.01	1
289	THR	HA	4.29	0.01	1
289	THR	HB	4.42	0.01	1

*Ambiguity index values are defined as in the Biological Magnetic Resonance Data Bank. 1: unique; 2: ambiguity of geminal atoms or geminal methyl proton groups; 3: aromatic atoms on opposite sides of symmetrical rings; 4: intraresidue ambiguity.

Table S1 Continuation. Assigned chemical shifts for the SH3 domain of human Caskin1 (20 mM K-phosphate, 100 mM KCl, 0.05% NaN_3, 0.1 mM TCEP, pH 7.2 at 10°C).

Residue number	Residue name	Atom name	Chemical shift (ppm)	Chemical shift error (ppm)	Ambiguity index*
289	THR	HG21	1.27	0.01	1
289	THR	HG22	1.27	0.01	1
289	THR	HG23	1.27	0.01	1
289	THR	CA	62.6	0.1	1
289	THR	CG	22.2	0.1	1
289	THR	CB	69.5	0.1	1
289	THR	N	115.5	0.1	1
290	LYS	H	7.39	0.01	1
290	LYS	HA	4.60	0.01	1
290	LYS	HB2	2.02	0.01	4
290	LYS	HB3	2.02	0.01	4
290	LYS	HG2	1.45	0.01	2
290	LYS	HG3	1.40	0.01	2
290	LYS	HD2	1.72	0.01	4
290	LYS	HD3	1.70	0.01	4
290	LYS	HE2	2.98	0.01	1
290	LYS	HE3	2.98	0.01	1
290	LYS	N	117.1	0.1	1
291	ASP	H	8.14	0.01	1
291	ASP	HA	4.60	0.01	1
291	ASP	HB2	2.71	0.01	2
291	ASP	HB3	2.63	0.01	2
291	ASP	CA	54.0	0.1	1
291	ASP	CB	41.6	0.1	1
291	ASP	N	116.5	0.1	1
292	TYR	H	9.04	0.01	1
292	TYR	HA	4.61	0.01	1
292	TYR	HB2	3.03	0.01	2
292	TYR	HB3	2.67	0.01	2
292	TYR	HD1	7.44	0.01	3
292	TYR	HD2	7.44	0.01	3
292	TYR	HE1	7.07	0.01	3
292	TYR	HE2	7.07	0.01	3
292	TYR	CA	58.8	0.1	1
292	TYR	CB	42.7	0.1	1
292	TYR	N	120.8	0.1	1
293	CYS	H	8.09	0.01	1
293	CYS	HA	4.42	0.01	1
293	CYS	HB2	2.75	0.01	2
293	CYS	HB3	2.7	0.01	2
293	CYS	CA	57.1	0.1	1

*Ambiguity index values are defined as in the Biological Magnetic Resonance Data Bank. 1: unique; 2: ambiguity of geminal atoms or geminal methyl proton groups; 3: aromatic atoms on opposite sides of symmetrical rings; 4: intraresidue ambiguity.

Table S1 Continuation. Assigned chemical shifts for the SH3 domain of human Caskin1 (20 mM K-phosphate, 100 mM KCl, 0.05% NaN_3, 0.1 mM TCEP, pH 7.2 at 10°C).

Residue number	Residue name	Atom name	Chemical shift (ppm)	Chemical shift error (ppm)	Ambiguity index*
293	CYS	CB	28.2	0.1	1
293	CYS	N	125.7	0.1	1
294	ASN	H	8.85	0.01	1
294	ASN	HA	4.81	0.01	1
294	ASN	HB2	2.43	0.01	2
294	ASN	HB3	2.99	0.01	2
294	ASN	HD21	7.32	0.01	2
294	ASN	HD22	7.12	0.01	2
294	ASN	CA	51.5	0.1	1
294	ASN	CB	38.6	0.1	1
294	ASN	N	124.7	0.1	1
294	ASN	ND2	110.8	0.1	1
295	ASN	HA	4.34	0.01	1
295	ASN	HB2	2.55	0.01	2
295	ASN	HB3	2.29	0.01	2
295	ASN	HD21	7.35	0.01	2
295	ASN	HD22	6.65	0.01	2
295	ASN	CA	54.5	0.1	1
295	ASN	CB	38.2	0.1	1
295	ASN	ND2	112.1	0.1	1
296	TYR	H	7.96	0.01	1
296	TYR	HA	4.45	0.01	1
296	TYR	HB2	3.21	0.01	2
296	TYR	HB3	2.94	0.01	2
296	TYR	HD1	7.88	0.01	3
296	TYR	HD2	7.88	0.01	3
296	TYR	HE1	7.16	0.01	3
296	TYR	HE2	7.16	0.01	3
296	TYR	CA	58.3	0.1	1
296	TYR	CB	38.1	0.1	1
296	TYR	N	116.0	0.1	1
297	ASP	H	7.13	0.01	1
297	ASP	HA	4.85	0.01	1
297	ASP	HB2	2.78	0.01	2
297	ASP	HB3	2.63	0.01	2
297	ASP	CA	52.1	0.1	1
297	ASP	CB	41.6	0.1	1
297	ASP	N	117.6	0.1	1
298	LEU	H	9.00	0.01	1
298	LEU	HA	4.35	0.01	1
298	LEU	HB2	1.78	0.01	2
298	LEU	N	126.1	0.1	1

*Ambiguity index values are defined as in the Biological Magnetic Resonance Data Bank. 1: unique; 2: ambiguity of geminal atoms or geminal methyl proton groups; 3: aromatic atoms on opposite sides of symmetrical rings; 4: intraresidue ambiguity.

Table S1 Continuation. Assigned chemical shifts for the SH3 domain of human Caskin1 (20 mM K-phosphate, 100 mM KCl, 0.05% NaN_3, 0.1 mM TCEP, pH 7.2 at 10°C).

Residue number	Residue name	Atom name	Chemical shift (ppm)	Chemical shift error (ppm)	Ambiguity index*
299	THR	H	8.49	0.01	1
299	THR	HA	4.79	0.01	1
299	THR	HG21	1.32	0.01	1
299	THR	HG22	1.32	0.01	1
299	THR	HG23	1.32	0.01	1
299	THR	CA	61.9	0.1	1
299	THR	CB	69.4	0.1	1
299	THR	CG	22.1	0.1	1
299	THR	N	108.2	0.1	1
300	SER	H	7.85	0.01	1
300	SER	HA	4.99	0.01	1
300	SER	HB2	4.29	0.01	2
300	SER	HB3	4.24	0.01	2
300	SER	CA	59.1	0.1	1
300	SER	CB	65.3	0.1	1
300	SER	N	118.2	0.1	1
301	LEU	H	9.33	0.01	1
301	LEU	HA	4.26	0.01	1
301	LEU	HB2	1.64	0.01	2
301	LEU	HB3	1.26	0.01	2
301	LEU	HD11	0.65	0.01	2
301	LEU	HD12	0.65	0.01	2
301	LEU	HD13	0.65	0.01	2
301	LEU	HD21	1.00	0.01	2
301	LEU	HD22	1.00	0.01	2
301	LEU	HD23	1.00	0.01	2
301	LEU	CD1	22.8	0.1	1
301	LEU	CD2	25.7	0.1	1
301	LEU	N	126.5	0.1	1
302	ASN	H	8.46	0.01	1
302	ASN	HA	5.02	0.01	1
302	ASN	HB2	2.72	0.01	1
302	ASN	HB3	2.72	0.01	1
302	ASN	HD21	7.56	0.01	2
302	ASN	HD22	6.91	0.01	2
302	ASN	CA	53.3	0.1	1
302	ASN	CB	38.6	0.1	1
302	ASN	N	124.1	0.1	1
302	ASN	ND2	112.4	0.1	1
303	VAL	H	9.29	0.01	1
303	VAL	HA	4.10	0.01	1
303	VAL	HB	2.15	0.01	1

*Ambiguity index values are defined as in the Biological Magnetic Resonance Data Bank. 1: unique; 2: ambiguity of geminal atoms or geminal methyl proton groups; 3: aromatic atoms on opposite sides of symmetrical rings; 4: intraresidue ambiguity.

Table S1 Continuation. Assigned chemical shifts for the SH3 domain of human Caskin1 (20 mM K-phosphate, 100 mM KCl, 0.05% NaN_3, 0.1 mM TCEP, pH 7.2 at 10°C).

Residue number	Residue name	Atom name	Chemical shift (ppm)	Chemical shift error (ppm)	Ambiguity index*
303	VAL	HG11	0.85	0.01	1
303	VAL	HG12	0.85	0.01	1
303	VAL	HG13	0.85	0.01	1
303	VAL	HG21	0.85	0.01	1
303	VAL	HG22	0.85	0.01	1
303	VAL	HG23	0.85	0.01	1
303	VAL	CA	61.9	0.1	1
303	VAL	CB	35.4	0.1	1
303	VAL	CG1	21.3	0.1	1
304	VAL	CG2	23.4	0.1	1
303	VAL	N	126.9	0.1	1
304	LYS	H	9.26	0.01	1
304	LYS	HA	4.88	0.01	1
304	LYS	HB2	1.70	0.01	4
304	LYS	HB3	1.70	0.01	4
304	LYS	HG2	1.42	0.01	1
304	LYS	HG3	1.42	0.01	1
304	LYS	HE2	2.99	0.01	1
304	LYS	HE3	2.99	0.01	1
304	LYS	N	130.3	0.1	1
305	ALA	H	9.00	0.01	1
305	ALA	HA	3.30	0.01	1
305	ALA	HB1	1.15	0.01	1
305	ALA	HB2	1.15	0.01	1
305	ALA	HB3	1.15	0.01	1
305	ALA	CA	54.0	0.1	1
305	ALA	CB	17.8	0.1	1
305	ALA	N	124.2	0.1	1
306	GLY	H	8.84	0.01	1
306	GLY	HA2	4.46	0.01	2
306	GLY	HA3	3.45	0.01	2
306	GLY	CA	44.9	0.1	1
306	GLY	N	112.2	0.1	1
307	ASP	H	8.57	0.01	1
307	ASP	HA	4.46	0.01	1
307	ASP	HB2	2.70	0.01	2
307	ASP	HB3	2.27	0.01	2
307	ASP	CA	55.8	0.1	1
307	ASP	CB	41.6	0.1	1
307	ASP	N	122.1	0.1	1
308	ILE	H	8.43	0.01	1
308	ILE	HA	4.58	0.01	1

*Ambiguity index values are defined as in the Biological Magnetic Resonance Data Bank. 1: unique; 2: ambiguity of geminal atoms or geminal methyl proton groups; 3: aromatic atoms on opposite sides of symmetrical rings; 4: intraresidue ambiguity.

Table S1 Continuation. Assigned chemical shifts for the SH3 domain of human Caskin1 (20 mM K-phosphate, 100 mM KCl, 0.05% NaN_3, 0.1 mM TCEP, pH 7.2 at 10°C).

Residue number	Residue name	Atom name	Chemical shift (ppm)	Chemical shift error (ppm)	Ambiguity index*
308	ILE	HB	1.92	0.01	1
308	ILE	HG12	1.75	0.01	2
308	ILE	HG13	1.34	0.01	2
308	ILE	HG21	0.75	0.01	1
308	ILE	HG22	0.75	0.01	1
308	ILE	HG23	0.75	0.01	1
308	ILE	HD11	0.80	0.01	1
308	ILE	HD12	0.80	0.01	1
308	ILE	HD13	0.80	0.01	1
308	ILE	CG1	27.3	0.1	1
308	ILE	CG2	17.5	0.1	1
308	ILE	CD1	14.2	0.1	1
308	ILE	N	120.4	0.1	1
309	ILE	H	9.29	0.01	1
309	ILE	HA	4.32	0.01	1
309	ILE	HG12	1.37	0.01	2
309	ILE	HG13	1.45	0.01	2
309	ILE	HG21	0.29	0.01	1
309	ILE	HG22	0.29	0.01	1
309	ILE	HG23	0.29	0.01	1
309	ILE	HD11	-0.08	0.01	1
309	ILE	HD12	-0.08	0.01	1
309	ILE	HD13	-0.08	0.01	1
309	ILE	CG1	27.5	0.1	1
309	ILE	CG2	18.3	0.1	1
309	ILE	CD1	13.6	0.1	1
309	ILE	N	128.4	0.1	1
310	THR	H	9.13	0.01	1
310	THR	HA	4.60	0.01	1
310	THR	HB	4.15	0.01	1
310	THR	HG21	1.13	0.01	1
310	THR	HG22	1.13	0.01	1
310	THR	HG23	1.13	0.01	1
310	THR	CA	63.5	0.1	1
310	THR	CB	69.0	0.1	1
310	THR	CG	22.1	0.1	1
310	THR	N	124.1	0.1	1
311	VAL	H	9.31	0.01	1
311	VAL	HA	4.00	0.01	1
311	VAL	HB	2.10	0.01	1
311	VAL	HG11	1.22	0.01	2
311	VAL	HG12	1.22	0.01	2

*Ambiguity index values are defined as in the Biological Magnetic Resonance Data Bank. 1: unique; 2: ambiguity of geminal atoms or geminal methyl proton groups; 3: aromatic atoms on opposite sides of symmetrical rings; 4: intraresidue ambiguity.

Table S1 Continuation. Assigned chemical shifts for the SH3 domain of human Caskin1 (20 mM K-phosphate, 100 mM KCl, 0.05% NaN_3, 0.1 mM TCEP, pH 7.2 at 10°C).

Residue number	Residue name	Atom name	Chemical shift (ppm)	Chemical shift error (ppm)	Ambiguity index*
311	VAL	HG13	1.22	0.01	2
311	VAL	HG21	0.65	0.01	2
311	VAL	HG22	0.65	0.01	2
311	VAL	HG23	0.65	0.01	2
311	VAL	CA	64.6	0.1	1
311	VAL	CB	31.3	0.1	1
311	VAL	CG1	23.1	0.1	1
311	VAL	CG2	22.8	0.1	1
311	VAL	N	129.4	0.1	1
312	LEU	H	9.04	0.01	1
312	LEU	HA	4.41	0.01	1
312	LEU	HB2	1.60	0.01	1
312	LEU	HB3	1.60	0.01	1
312	LEU	HG	1.22	0.01	1
312	LEU	HD11	0.81	0.01	2
312	LEU	HD12	0.81	0.01	2
312	LEU	HD13	0.81	0.01	2
312	LEU	HD21	-0.05	0.01	2
312	LEU	HD22	-0.05	0.01	2
312	LEU	HD23	-0.05	0.01	2
312	LEU	CG	29.8	0.1	1
312	LEU	CD1	21.5	0.1	1
312	LEU	CD2	23.4	0.1	1
312	LEU	N	128	0.1	1
313	GLU	H	7.64	0.01	1
313	GLU	HA	4.37	0.01	1
313	GLU	HB2	1.75	0.01	2
313	GLU	HB3	1.84	0.01	2
313	GLU	HG2	2.25	0.01	1
313	GLU	HG3	2.25	0.01	1
313	GLU	CA	55.3	0.1	1
313	GLU	CB	34.5	0.1	1
313	GLU	CG	35.8	0.1	1
313	GLU	N	114.3	0.1	1
314	GLN	H	8.39	0.01	1
314	GLN	HA	3.05	0.01	1
314	GLN	HB2	1.22	0.01	1
314	GLN	HB3	1.22	0.01	1
314	GLN	HG2	2.02	0.01	1
314	GLN	HG3	2.02	0.01	1
314	GLN	HE21	6.65	0.01	2
314	GLN	HE22	6.27	0.01	2

*Ambiguity index values are defined as in the Biological Magnetic Resonance Data Bank. 1: unique; 2: ambiguity of geminal atoms or geminal methyl proton groups; 3: aromatic atoms on opposite sides of symmetrical rings; 4: intraresidue ambiguity.

Table S1 Continuation. Assigned chemical shifts for the SH3 domain of human Caskin1 (20 mM K-phosphate, 100 mM KCl, 0.05% NaN_3, 0.1 mM TCEP, pH 7.2 at 10°C).

Residue number	Residue name	Atom name	Chemical shift (ppm)	Chemical shift error (ppm)	Ambiguity index*
314	GLN	CA	53.7	0.1	1
314	GLN	CB	29.6	0.1	1
314	GLN	CG	35.7	0.1	1
314	GLN	N	122.5	0.1	1
314	GLN	NE2	111.5	0.1	1
315	HIS	H	6.95	0.01	1
315	HIS	HA	4.88	0.01	1
315	HIS	HB2	3.31	0.01	2
315	HIS	HB3	3.28	0.01	2
315	HIS	CA	55.1	0.1	1
315	HIS	CB	32.6	0.1	1
315	HIS	N	121.2	0.1	1
316	PRO	HA	4.54	0.01	1
316	PRO	HB2	2.09	0.01	1
316	PRO	HB3	2.09	0.01	1
316	PRO	CA	64.8	0.1	1
316	PRO	CB	32.0	0.1	1
317	ASP	H	8.58	0.01	1
317	ASP	HA	4.52	0.01	1
317	ASP	HB2	2.96	0.01	2
317	ASP	HB3	2.68	0.01	2
317	ASP	CA	54.0	0.1	1
317	ASP	CB	39.8	0.1	1
317	ASP	N	117.7	0.1	1
318	GLY	H	8.25	0.01	1
318	GLY	HA2	4.17	0.01	2
318	GLY	HA3	3.63	0.01	2
318	GLY	CA	45.4	0.1	1
318	GLY	N	107.0	0.1	1
319	ARG	H	8.23	0.01	1
319	ARG	HA	4.53	0.01	1
319	ARG	HB2	1.42	0.01	1
319	ARG	HB3	1.42	0.01	1
319	ARG	HG2	1.92	0.01	2
319	ARG	HG3	1.80	0.01	2
319	ARG	HD2	2.90	0.01	1
319	ARG	HD3	2.90	0.01	1
319	ARG	CA	55.5	0.1	1
319	ARG	CB	29.1	0.1	1
319	ARG	CG	26.6	0.1	1
319	ARG	CD	43.5	0.1	1
319	ARG	N	123.5	0.1	1

*Ambiguity index values are defined as in the Biological Magnetic Resonance Data Bank. 1: unique; 2: ambiguity of geminal atoms or geminal methyl proton groups; 3: aromatic atoms on opposite sides of symmetrical rings; 4: intraresidue ambiguity.

Table S1 Continuation. Assigned chemical shifts for the SH3 domain of human Caskin1 (20 mM K-phosphate, 100 mM KCl, 0.05% NaN_3, 0.1 mM TCEP, pH 7.2 at 10°C).

Residue number	Residue name	Atom name	Chemical shift (ppm)	Chemical shift error (ppm)	Ambiguity index*
320	TRP	H	8.37	0.01	1
320	TRP	HA	5.19	0.01	1
320	TRP	HB2	2.95	0.01	2
320	TRP	HB3	2.00	0.01	2
320	TRP	HD1	7.00	0.01	4
320	TRP	HE1	10.08	0.01	1
320	TRP	HE3	7.16	0.01	4
320	TRP	HZ2	7.23	0.01	4
320	TRP	HZ3	6.86	0.01	4
320	TRP	HH2	7.41	0.01	4
320	TRP	CA	54.4	0.1	1
320	TRP	CB	31.8	0.1	1
320	TRP	N	126.8	0.1	1
320	TRP	NE1	127.5	0.1	1
321	LYS	H	8.83	0.01	1
321	LYS	HA	4.75	0.01	1
321	LYS	HB2	1.52	0.01	4
321	LYS	HB3	1.52	0.01	4
321	LYS	N	120.5	0.1	1
322	GLY	H	9.68	0.01	1
322	GLY	HA2	5.35	0.01	2
322	GLY	HA3	3.67	0.01	2
322	GLY	CA	45.4	0.1	1
322	GLY	N	114.2	0.1	1
323	CYS	H	8.96	0.01	1
323	CYS	HA	5.81	0.01	1
323	CYS	HB2	2.74	0.01	1
323	CYS	HB3	2.74	0.01	1
323	CYS	CA	55.2	0.1	1
323	CYS	CB	30.7	0.1	1
323	CYS	N	116.8	0.1	1
324	ILE	H	9.23	0.01	1
324	ILE	HA	4.47	0.01	1
324	ILE	HG21	0.88	0.01	1
324	ILE	HG22	0.88	0.01	1
324	ILE	HG23	0.88	0.01	1
324	ILE	HD11	0.89	0.01	1
324	ILE	HD12	0.89	0.01	1
324	ILE	HD13	0.89	0.01	1
324	ILE	N	121.9	0.1	1
325	HIS	H	9.13	0.01	1
325	HIS	HA	5.00	0.01	1

*Ambiguity index values are defined as in the Biological Magnetic Resonance Data Bank. 1: unique; 2: ambiguity of geminal atoms or geminal methyl proton groups; 3: aromatic atoms on opposite sides of symmetrical rings; 4: intraresidue ambiguity.

Table S1 Continuation. Assigned chemical shifts for the SH3 domain of human Caskin1 (20 mM K-phosphate, 100 mM KCl, 0.05% NaN_3, 0.1 mM TCEP, pH 7.2 at 10°C).

Residue number	Residue name	Atom name	Chemical shift (ppm)	Chemical shift error (ppm)	Ambiguity index*
325	HIS	HB2	3.08	0.01	1
325	HIS	HB3	3.08	0.01	1
325	HIS	CA	55.4	0.1	1
325	HIS	CB	29.7	0.1	1
325	HIS	N	127.3	0.1	1
326	ASP	H	8.34	0.01	1
326	ASP	HA	4.69	0.01	1
326	ASP	HB2	3.06	0.01	2
326	ASP	HB3	2.42	0.01	2
326	ASP	CA	53.0	0.1	1
326	ASP	CB	42.5	0.1	1
326	ASP	N	127.8	0.1	1
327	ASN	H	8.94	0.01	1
327	ASN	HA	4.40	0.01	1
327	ASN	HB2	2.86	0.01	1
327	ASN	HB3	2.86	0.01	1
327	ASN	HD21	7.70	0.01	2
327	ASN	HD22	7.03	0.01	2
327	ASN	CA	55.3	0.1	1
327	ASN	CB	38.6	0.1	1
327	ASN	N	123.8	0.1	1
327	ASN	ND2	112.6	0.1	1
328	ARG	H	8.66	0.01	1
328	ARG	HA	4.25	0.01	1
328	ARG	HB2	1.99	0.01	1
328	ARG	HB3	1.99	0.01	1
328	ARG	HG2	1.66	0.01	1
328	ARG	HG3	1.66	0.01	1
328	ARG	HD2	3.25	0.01	1
328	ARG	HD3	3.25	0.01	1
328	ARG	CA	58.6	0.1	1
328	ARG	CB	30.4	0.1	1
328	ARG	CG	28.5	0.1	1
328	ARG	CD	43.6	0.1	1
328	ARG	N	118.8	0.1	1
329	THR	H	7.57	0.01	1
329	THR	HA	4.33	0.01	1
329	THR	HB	4.31	0.01	1
329	THR	HG21	1.17	0.01	1
329	THR	HG22	1.17	0.01	1
329	THR	HG23	1.17	0.01	1
329	THR	CA	61.5	0.1	1

*Ambiguity index values are defined as in the Biological Magnetic Resonance Data Bank. 1: unique; 2: ambiguity of geminal atoms or geminal methyl proton groups; 3: aromatic atoms on opposite sides of symmetrical rings; 4: intraresidue ambiguity.

Table S1 Continuation. Assigned chemical shifts for the SH3 domain of human Caskin1 (20 mM K-phosphate, 100 mM KCl, 0.05% NaN_3, 0.1 mM TCEP, pH 7.2 at 10°C).

Residue number	Residue name	Atom name	Chemical shift (ppm)	Chemical shift error (ppm)	Ambiguity index*
329	THR	CB	70.6	0.1	1
329	THR	CG	21.5	0.1	1
329	THR	N	105.8	0.1	1
330	GLY	H	8.16	0.01	1
330	GLY	HA2	4.20	0.01	2
330	GLY	HA3	3.62	0.01	2
330	GLY	CA	45.7	0.1	1
330	GLY	N	110.2	0.1	1
331	ASN	H	7.68	0.01	1
331	ASN	HA	4.82	0.01	1
331	ASN	HB2	2.65	0.01	1
331	ASN	HB3	2.65	0.01	1
331	ASN	HD21	7.35	0.01	2
331	ASN	HD22	6.97	0.01	2
331	ASN	CA	52.4	0.1	1
331	ASN	CB	40.3	0.1	1
331	ASN	N	117.1	0.1	1
331	ASN	ND2	111.8	0.1	1
332	ASP	H	8.51	0.01	1
332	ASP	HA	5.35	0.01	1
332	ASP	HB2	2.47	0.01	1
332	ASP	HB3	2.47	0.01	1
332	ASP	CA	54.0	0.1	1
332	ASP	CB	43.2	0.1	1
332	ASP	N	120.4	0.1	1
333	ARG	H	8.81	0.01	1
333	ARG	HA	4.69	0.01	1
333	ARG	HB2	1.73	0.01	1
333	ARG	HB3	1.73	0.01	1
333	ARG	HD2	3.32	0.01	1
333	ARG	HD3	3.32	0.01	1
333	ARG	CA	55.6	0.1	1
333	ARG	CB	32.7	0.1	1
333	ARG	CD	43.6	0.1	1
333	ARG	N	122.6	0.1	1
334	VAL	H	8.59	0.01	1
334	VAL	HA	5.32	0.01	1
334	VAL	HB	1.89	0.01	1
334	VAL	HG11	0.86	0.01	2
334	VAL	HG12	0.86	0.01	2
334	VAL	HG13	0.86	0.01	2
334	VAL	HG21	0.37	0.01	2

*Ambiguity index values are defined as in the Biological Magnetic Resonance Data Bank. 1: unique; 2: ambiguity of geminal atoms or geminal methyl proton groups; 3: aromatic atoms on opposite sides of symmetrical rings; 4: intraresidue ambiguity.

Table S1 Continuation. Assigned chemical shifts for the SH3 domain of human Caskin1 (20 mM K-phosphate, 100 mM KCl, 0.05% NaN_3, 0.1 mM TCEP, pH 7.2 at 10°C).

Residue number	Residue name	Atom name	Chemical shift (ppm)	Chemical shift error (ppm)	Ambiguity index*
334	VAL	HG22	0.37	0.01	2
334	VAL	HG23	0.37	0.01	2
334	VAL	CA	59.8	0.1	1
334	VAL	CB	35.4	0.1	1
334	VAL	CG1	21.3	0.1	1
334	VAL	CG2	20.5	0.1	1
334	VAL	N	120.6	0.1	1
335	GLY	H	8.67	0.01	1
335	GLY	HA2	4.08	0.01	1
335	GLY	HA3	4.08	0.01	1
335	GLY	CA	45.5	0.1	1
335	GLY	N	111.2	0.1	1
336	TYR	H	8.93	0.01	1
336	TYR	HA	5.97	0.01	1
336	TYR	HB2	2.80	0.01	1
336	TYR	HB3	2.80	0.01	1
336	TYR	CA	58.1	0.1	1
336	TYR	CB	40.6	0.1	1
336	TYR	N	119.8	0.1	1
337	PHE	H	9.61	0.01	1
337	PHE	HA	5.04	0.01	1
337	PHE	HB2	3.02	0.01	2
337	PHE	HB3	2.63	0.01	2
337	PHE	HD1	7.59	0.01	3
337	PHE	HD2	7.59	0.01	3
337	PHE	HE1	7.75	0.01	3
337	PHE	HE2	7.75	0.01	3
337	PHE	CA	55.0	0.1	1
337	PHE	CB	39.6	0.1	1
337	PHE	N	119.5	0.1	1
339	SER	HA	4.08	0.01	1
339	SER	HB2	3.87	0.01	1
339	SER	HB3	3.87	0.01	1
339	SER	CA	60.3	0.1	1
339	SER	CB	60.0	0.1	1
340	SER	H	7.56	0.01	1
340	SER	HA	4.11	0.01	1
340	SER	HB2	3.95	0.01	2
340	SER	HB3	3.75	0.01	2
340	SER	CA	59.1	0.1	1
340	SER	CB	62.7	0.1	1
340	SER	N	114.9	0.1	1

*Ambiguity index values are defined as in the Biological Magnetic Resonance Data Bank. 1: unique; 2: ambiguity of geminal atoms or geminal methyl proton groups; 3: aromatic atoms on opposite sides of symmetrical rings; 4: intraresidue ambiguity.

Table S1 Continuation. Assigned chemical shifts for the SH3 domain of human Caskin1 (20 mM K-phosphate, 100 mM KCl, 0.05% NaN_3, 0.1 mM TCEP, pH 7.2 at 10°C).

Residue number	Residue name	Atom name	Chemical shift (ppm)	Chemical shift error (ppm)	Ambiguity index*
341	LEU	H	7.78	0.01	1
341	LEU	HA	4.23	0.01	1
341	LEU	HB2	2.06	0.01	2
341	LEU	HB3	1.75	0.01	2
341	LEU	HG	1.75	0.01	1
341	LEU	HD11	0.84	0.01	1
341	LEU	HD12	0.84	0.01	1
341	LEU	HD13	0.84	0.01	1
341	LEU	HD21	0.84	0.01	1
341	LEU	HD22	0.84	0.01	1
341	LEU	HD23	0.84	0.01	1
341	LEU	N	120.2	0.1	1
342	GLY	H	7.74	0.01	1
342	GLY	HA2	4.69	0.01	2
342	GLY	HA3	3.31	0.01	2
342	GLY	CA	44.8	0.1	1
342	GLY	N	103.1	0.1	1
343	GLU	H	8.73	0.01	1
343	GLU	HA	4.71	0.01	1
343	GLU	HB2	1.99	0.01	1
343	GLU	HB3	1.99	0.01	1
343	GLU	HG2	2.31	0.01	1
343	GLU	HG3	2.31	0.01	1
343	GLU	CA	54.0	0.1	1
343	GLU	CB	32.3	0.1	1
343	GLU	CG	34.9	0.1	1
343	GLU	N	122.5	0.1	1
344	ALA	H	8.75	0.01	1
344	ALA	HA	4.49	0.01	1
344	ALA	HB1	1.45	0.01	1
344	ALA	HB2	1.45	0.01	1
344	ALA	HB3	1.45	0.01	1
344	ALA	CA	53.2	0.1	1
344	ALA	CB	19.0	0.1	1
344	ALA	N	125.8	0.1	1
345	ILE	H	8.45	0.01	1
345	ILE	HA	4.15	0.01	1
345	ILE	HB	1.83	0.01	1
345	ILE	HG12	1.83	0.01	1
345	ILE	HG13	1.83	0.01	1
345	ILE	HG21	0.94	0.01	1
345	ILE	HG22	0.94	0.01	1

*Ambiguity index values are defined as in the Biological Magnetic Resonance Data Bank. 1: unique; 2: ambiguity of geminal atoms or geminal methyl proton groups; 3: aromatic atoms on opposite sides of symmetrical rings; 4: intraresidue ambiguity.

Table S1 Continuation. Assigned chemical shifts for the SH3 domain of human Caskin1 (20 mM K-phosphate, 100 mM KCl, 0.05% NaN_3, 0.1 mM TCEP, pH 7.2 at 10°C).

Residue number	Residue name	Atom name	Chemical shift (ppm)	Chemical shift error (ppm)	Ambiguity index*
345	ILE	HG23	0.94	0.01	1
345	ILE	HD11	0.94	0.01	1
345	ILE	HD12	0.94	0.01	1
345	ILE	HD13	0.94	0.01	1
345	ILE	N	121.9	0.1	1
346	VAL	H	7.78	0.01	1
346	VAL	HA	4.13	0.01	1
346	VAL	HB	2.08	0.01	1
346	VAL	HG11	0.90	0.01	1
346	VAL	HG12	0.90	0.01	1
346	VAL	HG13	0.90	0.01	1
346	VAL	HG21	0.90	0.01	1
346	VAL	HG22	0.90	0.01	1
346	VAL	HG23	0.90	0.01	1
346	VAL	CA	63.5	0.1	1
346	VAL	CB	33.6	0.1	1
346	VAL	CG1	21.7	0.1	1
346	VAL	CG2	24.0	0.1	1
346	VAL	N	128.6	0.1	1

*Ambiguity index values are defined as in the Biological Magnetic Resonance Data Bank. 1: unique; 2: ambiguity of geminal atoms or geminal methyl proton groups; 3: aromatic atoms on opposite sides of symmetrical rings; 4: intraresidue ambiguity.
