# Supplementary material for: Solution NMR Structure of the SH3 Domain of Human Caskin1 Validates the Lack of a Typical Peptide Binding Groove and Supports a Role in Lipid Mediator Binding
Source: Cells. 2021 Jan 16;10(1):173. doi: 10.3390/cells10010173 (PMC7830187; doi:10.3390/cells10010173)
Supplement: Supplementary file 1 [file cells-10-00173-s001.zip › caskin1-sh3-supplementary-201214/D_1292112096_val-report-full_P1.pdf]

Full wwPDB NMR Structure Validation Report (i)

Nov 11, 2020 – 09:24 PM GMT

PDB ID	:	7ATY
Title	:	Solution NMR structure of the SH3 domain of human Caskin1
Deposited on	:	2020-11-01

This is a Full wwPDB NMR Structure Validation Report.

This report is produced by the wwPDB biocuration pipeline after annotation of the structure.

We welcome your comments at *validation@mail.wwpdb.org*
A user guide is available at
https://www.wwpdb.org/validation/2017/NMRValidationReportHelp
with specific help available everywhere you see the (i) symbol.

CONFIDENTIAL VALIDATION REPORT

The following versions of software and data (see references (i)) were used in the production of this report:

Cyrange	:	Kirchner and Güntert (2011)
NmrClust	:	Kelley et al. (1996)
MolProbity	:	4.02b-467
Percentile statistics	:	20191225.v01 (using entries in the PDB archive December 25th 2019)
RCI	:	v_1n_11_5_13_A (Berjanski et al., 2005)
PANAV	:	Wang et al. (2010)
ShiftChecker	:	2.14.6
Ideal geometry (proteins)	:	Engh & Huber (2001)
Ideal geometry (DNA, RNA)	:	Parkinson et al. (1996)
Validation Pipeline (wwPDB-VP)	:	2.14.6

1 Overall quality at a glance

The following experimental techniques were used to determine the structure:
SOLUTION NMR

The overall completeness of chemical shifts assignment is 62%.

Percentile scores (ranging between 0-100) for global validation metrics of the entry are shown in the following graphic. The table shows the number of entries on which the scores are based.

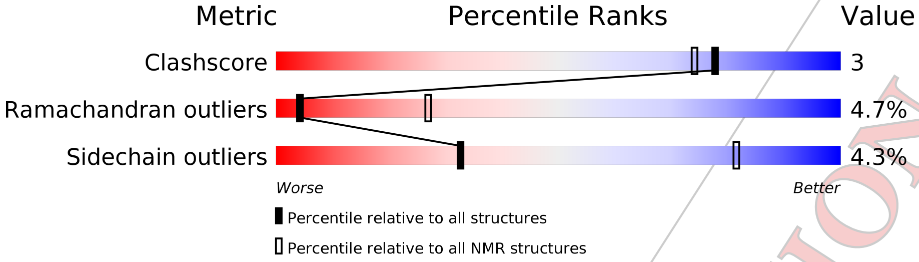

Metric	Whole archive (#Entries)	NMR archive (#Entries)
Clashscore	158937	12864
Ramachandran outliers	154571	11451
Sidechain outliers	154315	11428

The table below summarises the geometric issues observed across the polymeric chains and their fit to the experimental data. The red, orange, yellow and green segments indicate the fraction of residues that contain outliers for >=3, 2, 1 and 0 types of geometric quality criteria. A cyan segment indicates the fraction of residues that are not part of the well-defined cores, and a grey segment represents the fraction of residues that are not modelled. The numeric value for each fraction is indicated below the corresponding segment, with a dot representing fractions <=5%

Mol	Chain	Length	Quality of chain
1	A	67	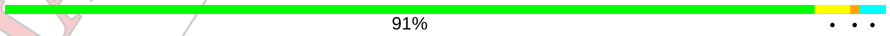

CONFIDENTIAL VALIDATION REPORT

2 Ensemble composition and analysis (i)

This entry contains 30 models. Model 13 is the overall representative, medoid model (most similar to other models). The authors have identified model 1 as representative, based on the following criterion: *lowest energy*.

The following residues are included in the computation of the global validation metrics.

Well-defined (core) protein residues			
**Well-defined core**	**Residue range (total)**	**Backbone RMSD (Å)**	**Medoid model**
1	A:3-A:67 (65)	0.77	13

Ill-defined regions of proteins are excluded from the global statistics.

Ligands and non-protein polymers are included in the analysis.

The models can be grouped into 6 clusters and 4 single-model clusters were found.

Cluster number	Models
1	3, 4, 9, 10, 13, 16, 18, 20, 21, 23, 26, 27, 28
2	2, 5, 7, 11, 12
3	1, 24
4	6, 29
5	14, 15
6	19, 30
Single-model clusters	8; 17; 22; 25

CONFIDENTIAL VALIDATION REPORT

3 Entry composition (i)

There is only 1 type of molecule in this entry. The entry contains 1026 atoms, of which 503 are hydrogens and 0 are deuteriums.

- Molecule 1 is a protein called Caskin-1.

Mol	Chain	Residues	Atoms						Trace
			Total	C	H	N	O	S	
1	A	67	1026	322	503	96	102	3	0

There are 4 discrepancies between the modelled and reference sequences:

Chain	Residue	Modelled	Actual	Comment	Reference
A	1	GLY	-	expression tag	UNP Q8WXD9
A	2	SER	-	expression tag	UNP Q8WXD9
A	3	HIS	-	expression tag	UNP Q8WXD9
A	4	MET	-	expression tag	UNP Q8WXD9

CONFIDENTIAL VALIDATION REPORT

4 Residue-property plots (i)

4.1 Average score per residue in the NMR ensemble

These plots are provided for all protein, RNA, DNA and oligosaccharide chains in the entry. The first graphic is the same as shown in the summary in section 1 of this report. The second graphic shows the sequence where residues are colour-coded according to the number of geometric quality criteria for which they contain at least one outlier: green = 0, yellow = 1, orange = 2 and red = 3 or more. Stretches of 2 or more consecutive residues without any outliers are shown as green connectors. Residues which are classified as ill-defined in the NMR ensemble, are shown in cyan with an underline colour-coded according to the previous scheme. Residues which were present in the experimental sample, but not modelled in the final structure are shown in grey.

- Molecule 1: Caskin-1

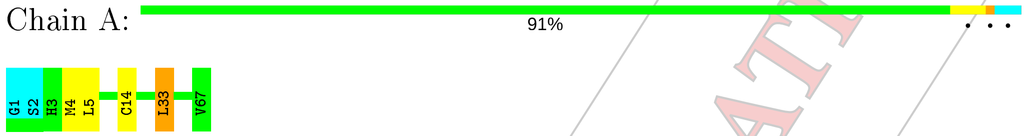

4.2 Scores per residue for each member of the ensemble

Colouring as in section 4.1 above.

4.2.1 Score per residue for model 1

- Molecule 1: Caskin-1

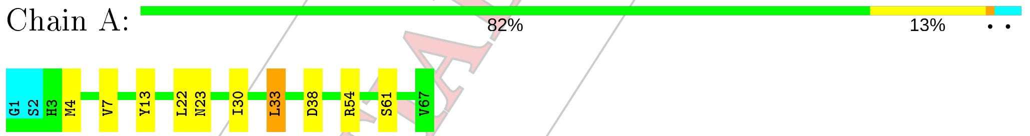

4.2.2 Score per residue for model 2

- Molecule 1: Caskin-1

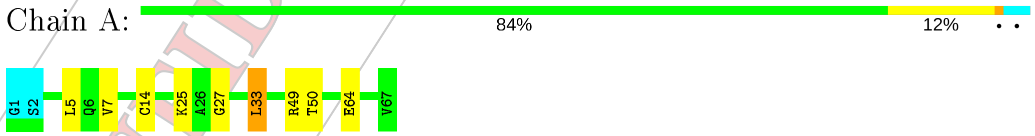

CONFIDENTIAL VALIDATION REPORT

4.2.3 Score per residue for model 3

- Molecule 1: Caskin-1

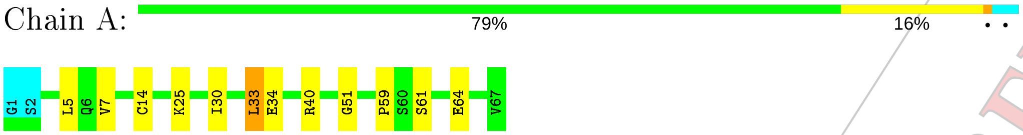

4.2.4 Score per residue for model 4

- Molecule 1: Caskin-1

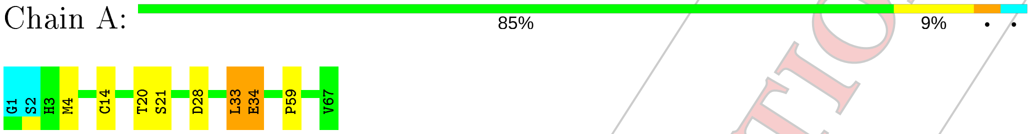

4.2.5 Score per residue for model 5

- Molecule 1: Caskin-1

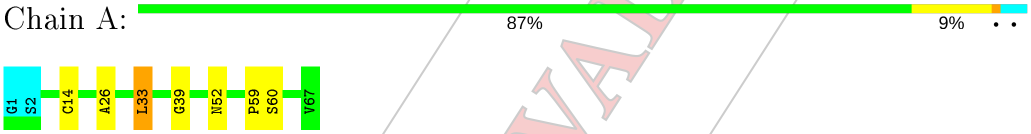

4.2.6 Score per residue for model 6

- Molecule 1: Caskin-1

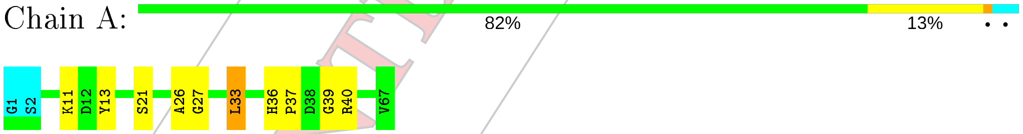

4.2.7 Score per residue for model 7

- Molecule 1: Caskin-1

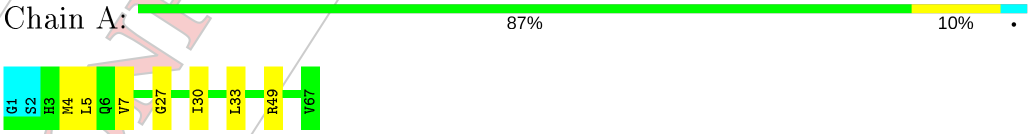

CONFIDENTIAL VALIDATION REPORT

4.2.8 Score per residue for model 8

• Molecule 1: Caskin-1

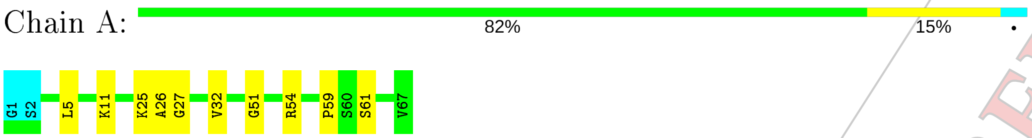

4.2.9 Score per residue for model 9

• Molecule 1: Caskin-1

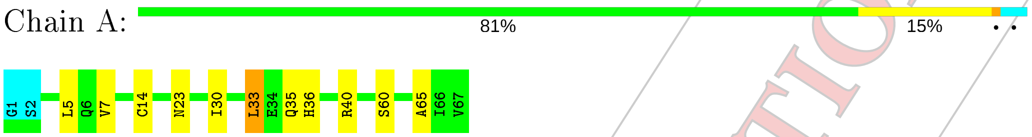

4.2.10 Score per residue for model 10

• Molecule 1: Caskin-1

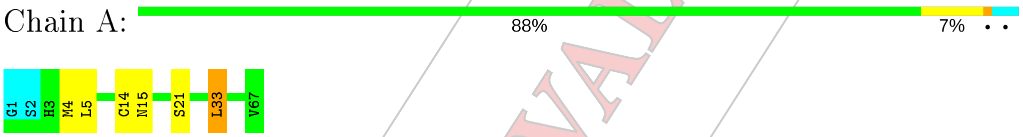

4.2.11 Score per residue for model 11

• Molecule 1: Caskin-1

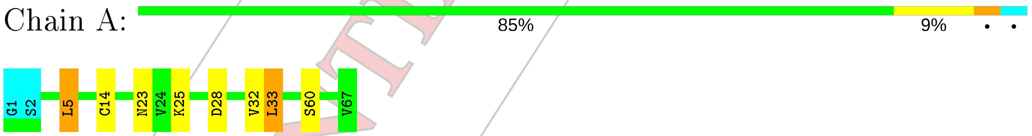

4.2.12 Score per residue for model 12

• Molecule 1: Caskin-1

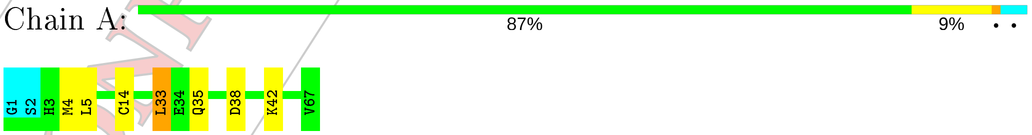

CONFIDENTIAL VALIDATION REPORT

4.2.13 Score per residue for model 13 (medoid)

- Molecule 1: Caskin-1

Chain A:

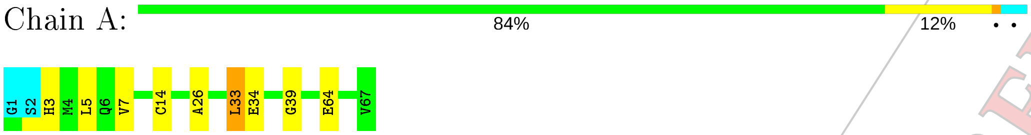

4.2.14 Score per residue for model 14

- Molecule 1: Caskin-1

Chain A:

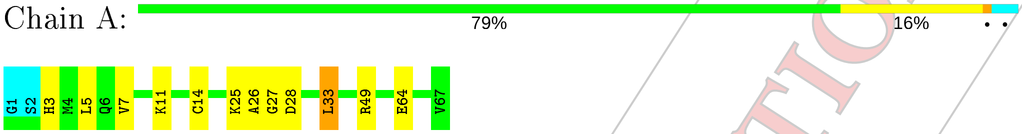

4.2.15 Score per residue for model 15

- Molecule 1: Caskin-1

Chain A:

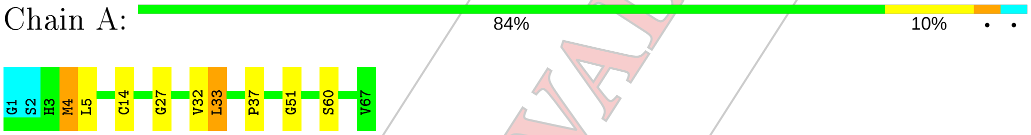

4.2.16 Score per residue for model 16

- Molecule 1: Caskin-1

Chain A:

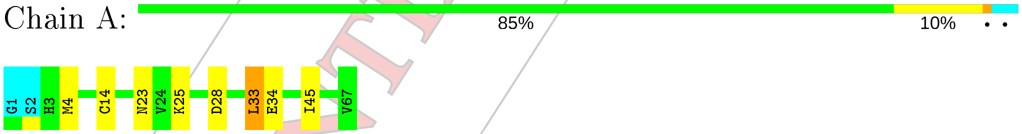

4.2.17 Score per residue for model 17

- Molecule 1: Caskin-1

Chain A:

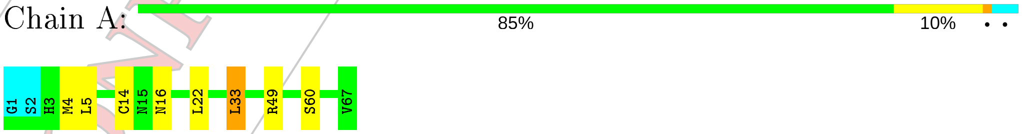

CONFIDENTIAL VALIDATION REPORT

4.2.18 Score per residue for model 18

- Molecule 1: Caskin-1

Chain A:

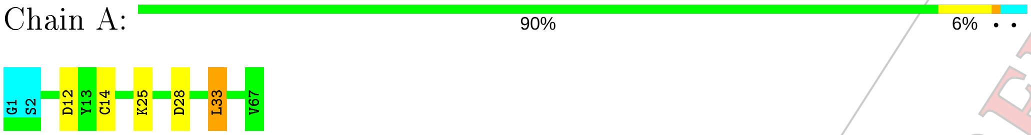

4.2.19 Score per residue for model 19

- Molecule 1: Caskin-1

Chain A:

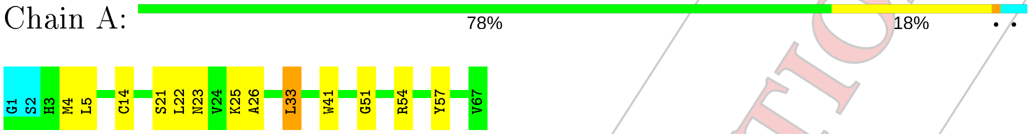

4.2.20 Score per residue for model 20

- Molecule 1: Caskin-1

Chain A:

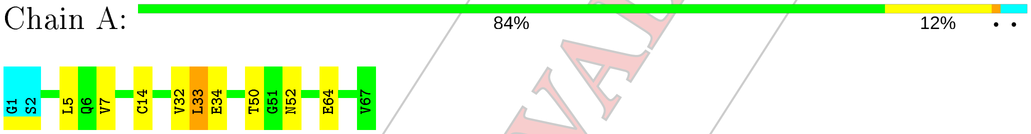

4.2.21 Score per residue for model 21

- Molecule 1: Caskin-1

Chain A:

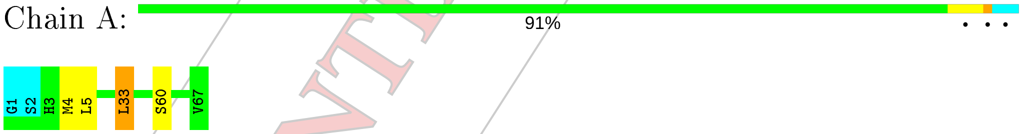

4.2.22 Score per residue for model 22

- Molecule 1: Caskin-1

Chain A:

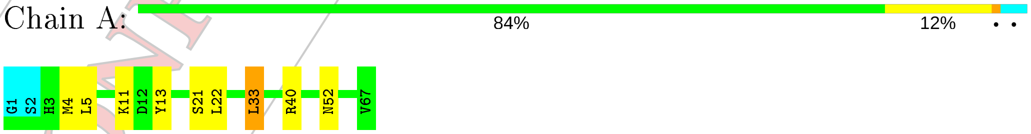

CONFIDENTIAL VALIDATION REPORT

4.2.23 Score per residue for model 23

- Molecule 1: Caskin-1

Chain A:

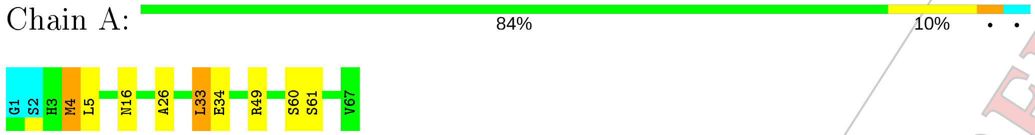

4.2.24 Score per residue for model 24

- Molecule 1: Caskin-1

Chain A:

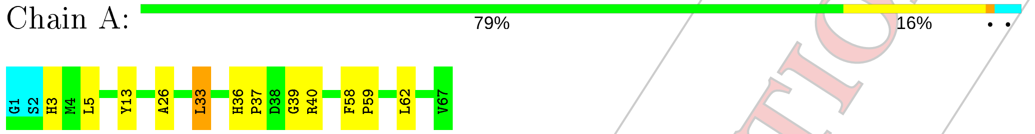

4.2.25 Score per residue for model 25

- Molecule 1: Caskin-1

Chain A:

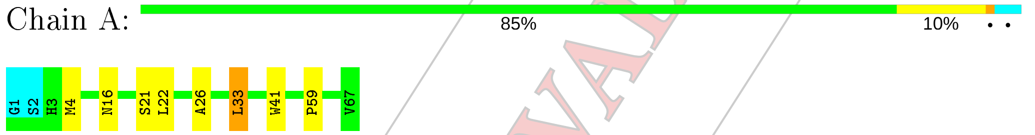

4.2.26 Score per residue for model 26

- Molecule 1: Caskin-1

Chain A:

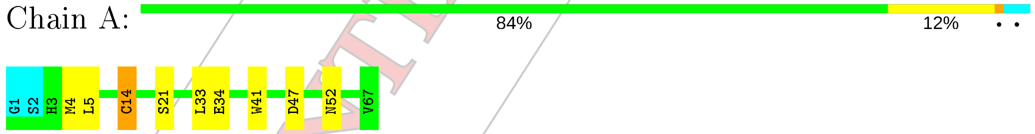

4.2.27 Score per residue for model 27

- Molecule 1: Caskin-1

Chain A:

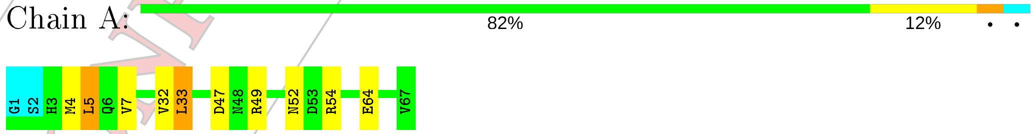

CONFIDENTIAL VALIDATION REPORT

4.2.28 Score per residue for model 28

- Molecule 1: Caskin-1

Chain A:

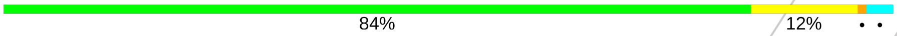

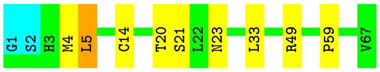

4.2.29 Score per residue for model 29

- Molecule 1: Caskin-1

Chain A:

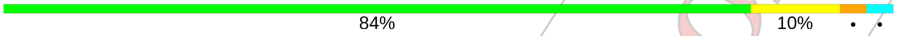

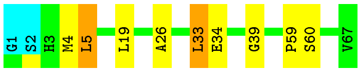

4.2.30 Score per residue for model 30

- Molecule 1: Caskin-1

Chain A:

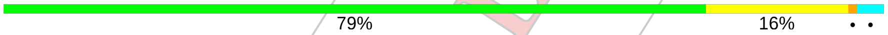

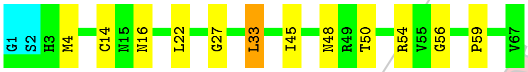

CONFIDENTIAL VALIDATION REPORT

5 Refinement protocol and experimental data overview (i)

The models were refined using the following method: *simulated annealing*.

Of the 100 calculated structures, 30 were deposited, based on the following criterion: *structures with the lowest energy*.

The following table shows the software used for structure solution, optimisation and refinement.

Software name	Classification	Version
ARIA	structure calculation	
ARIA	refinement	

The following table shows chemical shift validation statistics as aggregates over all chemical shift files. Detailed validation can be found in section 7 of this report.

Chemical shift file(s)	D_1292112096_cs_P1.cif.V1
Number of chemical shift lists	1
Total number of shifts	570
Number of shifts mapped to atoms	570
Number of unparsed shifts	0
Number of shifts with mapping errors	0
Number of shifts with mapping warnings	0
Assignment completeness (well-defined parts)	62%

No validations of the models with respect to experimental NMR restraints is performed at this time.

CONFIDENTIAL VALIDATION REPORT

6 Model quality (i)

6.1 Standard geometry (i)

There are no covalent bond-length or bond-angle outliers.

There are no bond-length outliers.

There are no bond-angle outliers.

There are no chirality outliers.

There are no planarity outliers.

6.2 Too-close contacts (i)

In the following table, the Non-H and H(model) columns list the number of non-hydrogen atoms and hydrogen atoms in each chain respectively. The H(added) column lists the number of hydrogen atoms added and optimized by MolProbity. The Clashes column lists the number of clashes averaged over the ensemble.

Mol	Chain	Non-H	H(model)	H(added)	Clashes
1	A	513	493	493	3±1
All	All	15390	14790	14790	88

The all-atom clashscore is defined as the number of clashes found per 1000 atoms (including hydrogen atoms). The all-atom clashscore for this structure is 3.

All unique clashes are listed below, sorted by their clash magnitude.

Atom-1	Atom-2	Clash(Å)	Distance(Å)	Models	
				Worst	Total
1:A:14:CYS:SG	1:A:21:SER:HB2	0.61	2.35	10	4
1:A:25:LYS:HG2	1:A:28:ASP:OD1	0.55	2.02	18	2
1:A:7:VAL:HA	1:A:64:GLU:O	0.54	2.03	3	6
1:A:54:ARG:HA	1:A:54:ARG:NE	0.54	2.18	27	1
1:A:25:LYS:HE3	1:A:28:ASP:OD1	0.52	2.04	16	1
1:A:4:MET:SD	1:A:33:LEU:HB3	0.52	2.44	22	1
1:A:11:LYS:N	1:A:26:ALA:HA	0.50	2.21	8	3
1:A:39:GLY:O	1:A:59:PRO:HA	0.49	2.06	5	2
1:A:23:ASN:OD1	1:A:54:ARG:HD2	0.49	2.08	1	1
1:A:25:LYS:HE2	1:A:50:THR:O	0.48	2.08	2	1
1:A:23:ASN:N	1:A:45:ILE:HG13	0.47	2.25	16	1
1:A:36:HIS:CE1	1:A:39:GLY:HA2	0.47	2.45	6	2
1:A:35:GLN:OE1	1:A:42:LYS:HG2	0.46	2.10	12	1
1:A:21:SER:HB3	1:A:57:TYR:O	0.45	2.10	19	1

Continued on next page...

CONFIDENTIAL VALIDATION REPORT

Continued from previous page...

Atom-1	Atom-2	Clash(Å)	Distance(Å)	Models	
				Worst	Total
1:A:33:LEU:HD13	1:A:33:LEU:H	0.45	1.70	20	1
1:A:11:LYS:HB3	1:A:13:TYR:CE1	0.45	2.47	22	1
1:A:7:VAL:CG2	1:A:30:ILE:HB	0.45	2.40	9	4
1:A:25:LYS:HG2	1:A:28:ASP:CG	0.45	2.32	11	1
1:A:33:LEU:N	1:A:33:LEU:HD13	0.44	2.28	17	16
1:A:34:GLU:HG2	1:A:41:TRP:CZ2	0.44	2.46	26	1
1:A:5:LEU:HG	1:A:32:VAL:CG2	0.44	2.43	27	4
1:A:33:LEU:HD13	1:A:33:LEU:N	0.44	2.27	15	9
1:A:47:ASP:HB3	1:A:52:ASN:H	0.43	1.72	27	1
1:A:23:ASN:OD1	1:A:54:ARG:HG2	0.43	2.13	19	1
1:A:4:MET:HA	1:A:32:VAL:O	0.43	2.14	15	1
1:A:47:ASP:HB3	1:A:52:ASN:N	0.43	2.28	26	1
1:A:5:LEU:HD13	1:A:5:LEU:N	0.42	2.28	29	1
1:A:25:LYS:CE	1:A:51:GLY:HA3	0.42	2.43	8	1
1:A:22:LEU:H	1:A:56:GLY:HA3	0.42	1.74	30	1
1:A:47:ASP:OD2	1:A:49:ARG:HB3	0.42	2.14	27	1
1:A:13:TYR:HD2	1:A:22:LEU:O	0.42	1.97	1	1
1:A:21:SER:O	1:A:22:LEU:HB2	0.42	2.13	25	2
1:A:23:ASN:H	1:A:45:ILE:HG13	0.41	1.75	16	1
1:A:45:ILE:HB	1:A:54:ARG:HB2	0.41	1.92	30	1
1:A:58:PHE:CD2	1:A:62:LEU:HB2	0.41	2.51	24	1
1:A:36:HIS:N	1:A:37:PRO:HD3	0.41	2.31	6	1
1:A:7:VAL:HG12	1:A:65:ALA:HA	0.41	1.93	9	1
1:A:36:HIS:HB2	1:A:40:ARG:O	0.41	2.15	9	1
1:A:33:LEU:O	1:A:41:TRP:HB3	0.41	2.16	19	2
1:A:37:PRO:HD2	1:A:40:ARG:O	0.41	2.16	24	1
1:A:36:HIS:NE2	1:A:39:GLY:HA2	0.40	2.32	24	1
1:A:13:TYR:CE2	1:A:21:SER:HB3	0.40	2.51	6	1
1:A:5:LEU:N	1:A:5:LEU:HD13	0.40	2.32	28	1
1:A:33:LEU:H	1:A:33:LEU:HD13	0.40	1.75	29	1

6.3 Torsion angles (i)

6.3.1 Protein backbone (i)

In the following table, the Percentiles column shows the percent Ramachandran outliers of the chain as a percentile score with respect to all PDB entries followed by that with respect to all NMR entries. The Analysed column shows the number of residues for which the backbone conformation was analysed and the total number of residues.

CONFIDENTIAL VALIDATION REPORT

Mol	Chain	Analysed	Favoured	Allowed	Outliers	Percentiles	
1	A	64/67 (96%)	51±2 (79±3%)	10±2 (16±4%)	3±1 (5±2%)	4	27
All	All	1920/2010 (96%)	1522 (79%)	307 (16%)	91 (5%)	4	27

All 19 unique Ramachandran outliers are listed below. They are sorted by the frequency of occurrence in the ensemble.

Mol	Chain	Res	Type	Models (Total)
1	A	4	MET	17
1	A	14	CYS	16
1	A	60	SER	8
1	A	27	GLY	7
1	A	26	ALA	7
1	A	59	PRO	7
1	A	49	ARG	6
1	A	61	SER	4
1	A	3	HIS	3
1	A	51	GLY	3
1	A	38	ASP	2
1	A	52	ASN	2
1	A	50	THR	2
1	A	22	LEU	2
1	A	16	ASN	1
1	A	37	PRO	1
1	A	39	GLY	1
1	A	15	ASN	1
1	A	35	GLN	1

6.3.2 Protein sidechains (i)

In the following table, the Percentiles column shows the percent sidechain outliers of the chain as a percentile score with respect to all PDB entries followed by that with respect to all NMR entries. The Analysed column shows the number of residues for which the sidechain conformation was analysed and the total number of residues.

Mol	Chain	Analysed	Rotameric	Outliers	Percentiles	
1	A	56/57 (98%)	54±1 (96±2%)	2±1 (4±2%)	33	81
All	All	1680/1710 (98%)	1608 (96%)	72 (4%)	33	81

All 16 unique residues with a non-rotameric sidechain are listed below. They are sorted by the frequency of occurrence in the ensemble.

CONFIDENTIAL VALIDATION REPORT

Mol	Chain	Res	Type	Models (Total)
1	A	33	LEU	29
1	A	5	LEU	20
1	A	40	ARG	3
1	A	23	ASN	3
1	A	16	ASN	3
1	A	34	GLU	2
1	A	20	THR	2
1	A	25	LYS	2
1	A	12	ASP	1
1	A	13	TYR	1
1	A	4	MET	1
1	A	19	LEU	1
1	A	52	ASN	1
1	A	54	ARG	1
1	A	48	ASN	1
1	A	28	ASP	1

6.3.3 RNA (i)

There are no RNA molecules in this entry.

6.4 Non-standard residues in protein, DNA, RNA chains (i)

There are no non-standard protein/DNA/RNA residues in this entry.

6.5 Carbohydrates (i)

There are no monosaccharides in this entry.

6.6 Ligand geometry (i)

There are no ligands in this entry.

6.7 Other polymers (i)

There are no such molecules in this entry.

6.8 Polymer linkage issues (i)

There are no chain breaks in this entry.

CONFIDENTIAL VALIDATION REPORT

7 Chemical shift validation (i)

The completeness of assignment taking into account all chemical shift lists is 62% for the well-defined parts and 62% for the entire structure.

7.1 Chemical shift list 1

File name: D_1292112096_cs_P1.cif.V1

Chemical shift list name: *starch_output*

7.1.1 Bookkeeping (i)

The following table shows the results of parsing the chemical shift list and reports the number of nuclei with statistically unusual chemical shifts.

Total number of shifts	570
Number of shifts mapped to atoms	570
Number of unparsed shifts	0
Number of shifts with mapping errors	0
Number of shifts with mapping warnings	0
Number of shift outliers (ShiftChecker)	0

7.1.2 Chemical shift referencing (i)

The following table shows the suggested chemical shift referencing corrections.

Nucleus	# values	Correction ± precision, *ppm*	Suggested action
$^{13}C_{\alpha}$	54	-0.24 ± 0.22	None needed (< 0.5 ppm)
$^{13}C_{\beta}$	47	0.19 ± 0.25	None needed (< 0.5 ppm)
$^{13}C'$	0	—	None (insufficient data)
^{15}N	61	-0.52 ± 0.90	None needed (imprecise)

7.1.3 Completeness of resonance assignments (i)

The following table shows the completeness of the chemical shift assignments for the well-defined regions of the structure. The overall completeness is 62%, i.e. 490 atoms were assigned a chemical shift out of a possible 789. 0 out of 10 assigned methyl groups (LEU and VAL) were assigned stereospecifically.

	Total	^{1}H	^{13}C	^{15}N
Backbone	239/321 (74%)	125/128 (98%)	53/130 (41%)	61/63 (97%)
Sidechain	232/402 (58%)	179/234 (76%)	46/146 (32%)	7/22 (32%)

Continued on next page...

CONFIDENTIAL VALIDATION REPORT

Continued from previous page...

	Total	^{1}H	^{13}C	^{15}N
Aromatic	19/66 (29%)	18/35 (51%)	0/27 (0%)	1/4 (25%)
Overall	490/789 (62%)	322/397 (81%)	99/303 (33%)	69/89 (78%)

The following table shows the completeness of the chemical shift assignments for the full structure. The overall completeness is 62%, i.e. 495 atoms were assigned a chemical shift out of a possible 802. 0 out of 10 assigned methyl groups (LEU and VAL) were assigned stereospecifically.

	Total	^{1}H	^{13}C	^{15}N
Backbone	241/331 (73%)	126/132 (95%)	54/134 (40%)	61/65 (94%)
Sidechain	235/405 (58%)	181/236 (77%)	47/147 (32%)	7/22 (32%)
Aromatic	19/66 (29%)	18/35 (51%)	0/27 (0%)	1/4 (25%)
Overall	495/802 (62%)	325/403 (81%)	101/308 (33%)	69/91 (76%)

7.1.4 Statistically unusual chemical shifts (i)

There are no statistically unusual chemical shifts.

7.1.5 Random Coil Index (RCI) plots (i)

The image below reports *random coil index* values for the protein chains in the structure. The height of each bar gives a probability of a given residue to be disordered, as predicted from the available chemical shifts and the amino acid sequence. A value above 0.2 is an indication of significant predicted disorder. The colour of the bar shows whether the residue is in the well-defined core (black) or in the ill-defined residue ranges (cyan), as described in section 2 on ensemble composition.

Random coil index (RCI) for chain A:

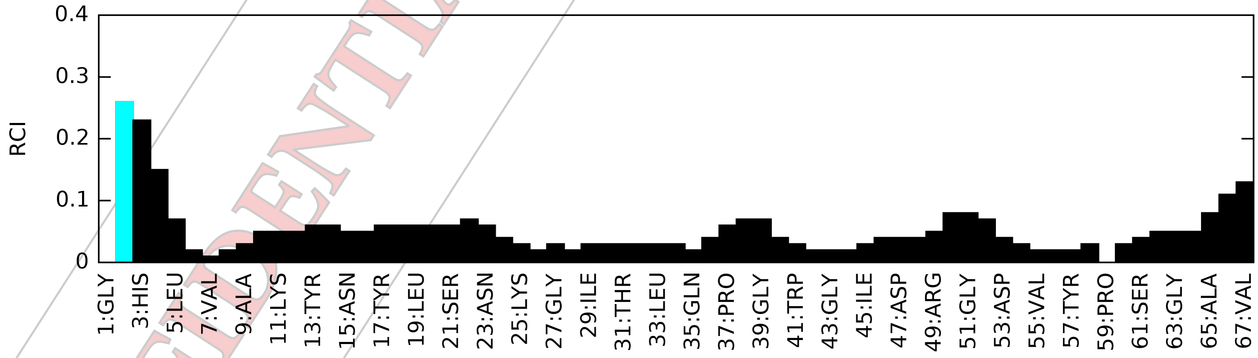

CONFIDENTIAL VALIDATION REPORT
